# Enhancing Differentiation of Oxygenated Organic Aerosol: A Machine Learning Approach to Distinguish Local and Transboundary Pollution

**DOI:** 10.1021/acsestair.4c00331

**Published:** 2025-04-15

**Authors:** Lu Lei, Wei Xu, Chunshui Lin, Baihua Chen, Kirsten N. Fossum, Darius Ceburnis, Colin O’Dowd, Jurgita Ovadnevaite

**Affiliations:** † School of Natural Sciences, Ryan Institute’s Centre for Climate & Air Pollution Studies, University of Galway, Galway, H91 CF50 Ireland; ‡ Center for Excellence in Regional Atmospheric Environment, Institute of Urban Environment, Chinese Academy of Sciences, Xiamen, 361021 China; § State Key Laboratory of Loess and Quaternary Geology and Key Laboratory of Aerosol Chemistry and Physics, Institute of Earth Environment, Chinese Academy of Sciences, Xi’an, 710061 China

**Keywords:** Oxygenated organic aerosol, Source apportionment, Rolling PMF, Machine learning, Urban air pollution

## Abstract

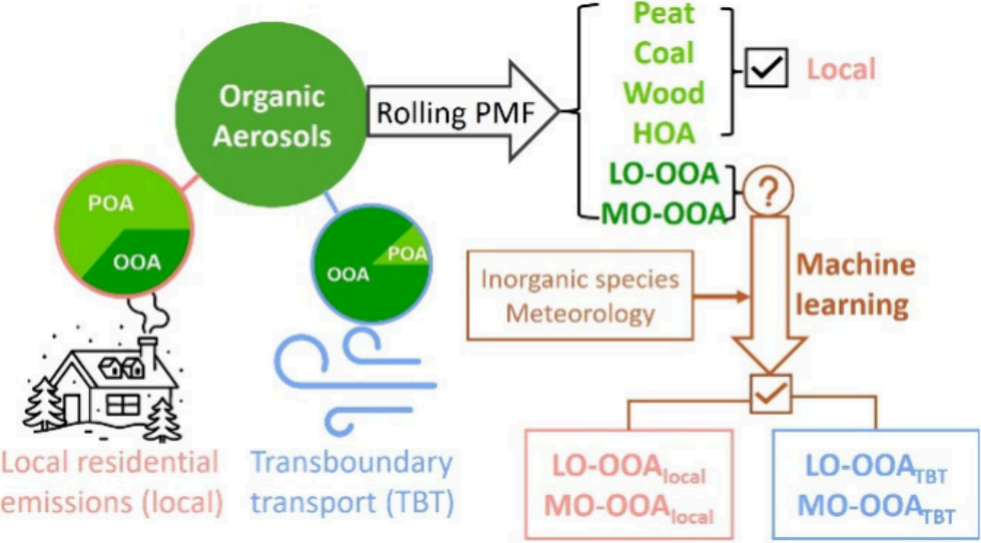

Accurate source apportionment of particulate matter (PM), especially of organic aerosol (OA), is crucial for targeted mitigation efforts. Positive Matrix Factorization (PMF) is powerful in source attribution of primary OA (POA); however, it often struggles to differentiate sources of oxygenated OA (OOA) due to their similar chemical profiles. In this study, a support vector regression machine learning (ML) model was developed to enhance the OOA source apportionment in Dublin from 2016 to 2023. Rolling PMF analysis identified four POA factors and differentiated OOA into less- and more-oxidized (LO-OOA and MO-OOA), highlighting the significant role of the OOA (47–74% of total OA). The ML model further distinguished locally produced OOA (LO-OOA_local_ and MO-OOA_local_) from transboundary transport OOA and exhibited robust performance across different pollution scenarios. The relative importance analysis revealed that LO-OOA_local_ was more impacted by fossil fuel emissions like hydrocarbon-like OA (20%) and coal (14%), whereas MO-OOA_local_ was most influenced by LO-OOA (17%), providing insights into their sources and formation mechanisms. During a mixed pollution episode, the results show that despite the significant contribution of transboundary transport, local heating emissions were more critical sources of OA, with local OA accounting for 68% of total OA and reaching 78% during heating hours. These findings highlight the ongoing need to reduce local emissions to achieve cleaner air in Dublin. The ML model’s ability to quantitatively separate local and transboundary OOA offers invaluable insights for future air quality regulations.

## Abstract 2

Machine learning is applied to distinguish local and transboundary contributions to oxygenated organic aerosol in Dublin, providing quantitative insights into air quality regulations.

## 1 Introduction

Particulate matter (PM) suspended in the atmosphere significantly impairs air quality and influences the regional and global climate directly and indirectly, introducing large uncertainties in radiative forcing estimation.^1,2^ Additionally, PM adversely impacts human health, causing millions of premature deaths every year.^3−5^ Organic aerosol (OA), constituting 20–90% of submicron PM,^6^ has been found to be more toxic than inorganic species such as nitrate and sulfate, raising greater health concerns.^7−9^ However, OA remains the least understood component due to the high complexity in its composition, sources, physicochemical properties, and formation pathways.^10^ Positive Matrix Factorization (PMF) has been widely used as a powerful tool achieving meaningful OA source apportionment for better understanding of its origins.^6,11,12^ While PMF analysis can provide valuable information on primary OA (POA) sources, especially when combined with the multilinear engine algorithm (ME-2), it faces significant challenges in distinguishing oxygenated OA (OOA) from different sources/processes. This is mainly because OOA loses original source signatures as it undergoes atmospheric oxidation, leading to increasingly similar chemical profiles.^13^ When measured by aerosol mass spectrometer, OOA tends to fragment into only a few main ions, such as CO_2_^+^ and C_2_H_3_O^+^,^6,14^ posing challenges for source apportionment, particularly for unit mass resolution data. As a result, OOA factors are typically classified by their relative oxidation degree, e.g., less oxidized OOA (LO-OOA), which serves as a surrogate of fresher, less-aged secondary OA (SOA) and more oxidized OOA (MO-OOA), a proxy of regional, more-aged SOA.^11,15^ However, this differentiation provides limited information on their sources and formation processes.^12^ Additionally, the PMF model has inherent limitations. Most importantly, it assumes linear relationships between sources and observations,^16^ which may not always be true for complex atmospheric processes where nonlinear interactions and secondary formation are involved, introducing uncertainty and ambiguity in OA source apportionment.

In recent decades, machine learning (ML) algorithms have been widely applied in atmospheric science across various topics.^17,18^ For example, extensive studies have utilized machine learning methods on air quality forecasting and prediction,^19,20^ evaluating the relative influences on PM concentrations from emissions and meteorological conditions,^21,22^ investigating the source impacts on visibility,^23^ and exploring the nonlinear relationship among volatile organic compounds (VOCs), PM_2.5_, and O_3_.^24^ Additionally, the applications of machine learning algorithms for PM source apportionment are also rapidly increasing and are less computationally expensive. For instance, Qiao et al.^25^ utilized the decision tree algorithm to attribute atmospheric oxygenated organic molecules to their precursors. Heikkinen et al.^26^ deployed the k-means clustering method to identify OA subtypes in a remote boreal forest site in Southern Finland, where the OA mass concentration remains low and traditional PMF is ineffective. Pande et al.^27^ developed a two-step machine learning method, combining a multinomial logistic classifier and an ensemble regression model for rapid OA source apportionment. The results showed high classification accuracy and broad qualitative agreement with PMF analysis. These extensive and successful applications of machine learning algorithms on air pollution data analysis demonstrate their unique advantages in capturing nonlinear effects and discerning subtle patterns within multidimensional data sets. This makes machine learning methods well-suited to enhance the apportionment of the OOA source, linking the OOA to more specific sources.

Ireland once experienced extreme air pollution in the 1980s primarily caused by coal combustion.^28^ Although a series of bans on smoky coal burning since 1990 have significantly alleviated the air pollution in Ireland, Lin et al.^29^ pointed out that extreme air pollution events were still regularly occurring in Dublin during the residential heating season. The OA source apportionment revealed that these extreme air pollution events primarily result from disproportionately high emissions from domestic solid fuel burning within the broader Dublin urban area (referred to as local). Importantly, in addition to significantly elevated primary species, OOA also shows substantial increase during these local events.^30^ On the other hand, air pollution events resulting from transboundary transport are also often observed in Ireland, with OA being overwhelmingly dominated by OOA.^31,32^ In other words, the OOA always plays a crucial role in causing air pollution in Dublin. However, as highlighted above, traditional PMF analysis is often insufficient for OOA attribution, leading to significant ambiguity in its source identification and quantification of relative contributions, which hinders the implementation of more targeted control measures to reduce OA pollution.

In this study, the capabilities of machine learning in enhancing source apportionment of OOA were explored. A supervised machine learning model was developed in combination with rolling-PMF analysis, trained with a carefully selected data set from pollution events dominated by local emissions. Model performance was evaluated using statistical metrics and further validated with two distinct pollution episodes: one dominated by local sources and one dominated by transboundary sources. The ML model was also applied to a mixed pollution event with overlapping impacts from local and transboundary sources to quantify their relative contributions on the OOA. Importantly, none of the three pollution episodes selected for case studies were included in the training data set, ensuring valid evaluations.

## 2 Materials and Methods

### 2.1 Instruments and Data Collection

The measurements of chemical-speciated submicrometer PM (i.e., PM_1_, particulate matter with a diameter less than 1 μm) were conducted at the urban background site in Dublin, Ireland, from August 2016 to December 2023. Detailed introduction of the sampling site can be found in Lin et al.^30^ An Aerodyne Quadrupole Aerosol Chemical Speciation Monitor (Q-ACSM, Aerodyne Research Inc., USA) was deployed for the measurements of nonrefractory species in PM_1_ (NR-PM_1_) including Organic Aerosol (OA), sulfate (SO_4_), nitrate (NO_3_), ammonium (NH_4_), and chloride (Cl). More details about the sampling protocol of Q-ACSM can be found in previous studies.^33,34^ The Q-ACSM deployed in this study was regularly calibrated following standard calibration protocols to ensure data quality.^34^ Additionally, the chemical composition collection efficiency^35^ was applied to NR-PM_1_ species consistently across all years. More details on NR-PM_1_ measurements in Dublin are summarized in S1.1, including deployed Q-ACSM instruments, data coverage, NR-PM_1_ concentration ranges (Tables S1–S2), and also instrument stability (Figure S1a). An Aethalometer (model AE33 from Magee Scientific) was also deployed to measure the optical attenuation at 7 wavelengths. The mass concentration of equivalent black carbon (eBC) was then retrieved using attenuation at 880 nm with a standard mass absorption cross-section value of 7.77 m^2^ g^–1^. In addition, a Scanning Mobility Particle Sizer (SMPS) was collocated to measure the PM number concentration and size distribution in the range 10–500 nm. Hourly meteorological parameters, including wind speed (WS), wind direction (WD), relative humidity (RH), and ambient temperature (*T*) were from Dublin Airport (https://www.met.ie/climate/available-data/historical-data). To ensure consistency of temporal resolution across all data sets, hourly mean values were used for further analysis.

A rigorous flagging process was implemented to remove periods with operational issues or anomalies, ensuring data set reliability. To validate the data quality and accuracy of the data set from Dublin site in this study, the reconstructed PM_1_ (= NR-PM_1_ + eBC) was compared with the collocated SMPS measurements, with the PM_1_ mass concentration converted into volume concentration by dividing the mass concentrations of each species by their respective densities.^36^ Additionally, PM_2.5_ mass concentration measurements from the Rathmines monitoring station located around 3 km from the Dublin site were used as an external comparison data set. The intercomparisons showed high correlation coefficients ranging from 0.83 to 0.97, associated with reasonable slopes (0.84 for PM_2.5_/PM_1_ and 1.15 for PM_1_/SMPS, respectively), which validated the Dublin data set (Figure S1b-c). Additionally, the good agreement with PM_2.5_ measurements from Rathmines confirmed that the impacts from nearby local point sources are insignificant at the Dublin sampling site.

### 2.2 Rolling Positive Matrix Factorization (Rolling PMF)

The raw data set collected by Q-ACSM was processed using standard data analysis software (version 1.6.1.1) based on Igor Pro (Wavemetrics Inc.) to obtain the mass concentration of NR-PM_1_ species. The concentration and uncertainty matrices of OA were also exported from this software and averaged to hourly resolution, serving as inputs of PMF analysis. To enhance the separation of OA factors originating from different sources, mass profiles of peat (OA from peat burning), wood (OA from wood burning), and coal (OA from coal burning) obtained from burning experiments^37−39^ and the hydrocarbon-like OA (HOA) profile from AMS UMR MS database (https://cires1.colorado.edu/jimenez-group/AMSsd/), which was measured in Paris,^40^ were used as reference profiles to constrain the POA factors using ME-2 algorithm under Source Finder pro (SoFi-pro). Taking the dynamic nature of OA factors into consideration, the advanced rolling-PMF analysis, which is able to dynamically adjust the mass profiles over time,^41^ was performed, with a rolling time-window of 14 days and shifting step of 1 day, respectively. In addition, segmented PMF analysis and the “limits” a-value approach were applied to further account for potential source variability over time. The constraining strategies and bootstrap resampling applied to the data set in this study are consistent with our previous studies in Dublin.^39,42^ More details are provided in S1.2, and the criteria for PMF run selection are summarized in Table S3.

### 2.3 Machine Learning Model

#### 2.3.1 Training Data Set Selection

A supervised machine learning model was developed (Figure S2) to enhance the differentiation of the OOA into local and transboundary sources in this study. Pollution events dominated by local emissions were selected as the model training data set to establish relationships between OOA from local sources and their corresponding predictor variables. To ensure the reliability of selected local air pollution events, a set of screening criteria (Table S4) was implemented to exclude impacts from regional sources: (1) Previous studies have demonstrated that, in Dublin, the air pollution events caused by local residential heating emissions mainly occur in cold months,^42^ so only data from October to March were selected for model training; (2) Data points when OA mass concentration was lower than 0.5 μg m^–3^ were excluded to avoid high uncertainty associated with lower signal-to-noise ratios; (3) The mass fraction of POA factors to total OA was constrained to be higher than OOA (POA fraction >50%) to ensure the dominance of local emissions; (4) The mass ratio of OA to NO_3_ was constrained to be higher than 2 to exclude significant impacts from regional transport, which features high contribution of NO_3_;^32^ and (5) Data points were selected only when WS was below 5 m s^–1^, as stagnant meteorological conditions favored extreme pollution events. The thresholds of filtering criteria were chosen to balance the selection of representative local events, while ensuring adequate data for reliable model training. Sensitivity tests, as shown in Figure S3a, confirmed that the model effectively captures the characteristics of local emissions without overly dependent on specific thresholds. As a result, ∼5% of the entire data set (2733 out of 56176 data points) was selected for model training and feature selection.

#### 2.3.2 Model Selection, Optimization, and Evaluation

A support vector regression (SVR) model was chosen in this study to predict the OOA from local sources due to its well-documented advantages in handling nonlinear relationships, robustness even with relatively small data sets, and its resistance to overfitting.^43^ The choice of SVR was further validated through comparisons with other machine learning algorithms (Figure S3b). The selection of model predictors was optimized to ensure the best model performance (Figure S4a,b), with further details provided in the Supplement (S1.3). As summarized in Table S4, primary species (POA, eBC, and Cl) were included as model inputs as they tend to spike concurrently with OOA during pollution events dominated by local emissions.^29,30^ WD was also incorporated in the model, as it provides information about air mass origins, which is crucial for understanding local pollution dynamics. Hour of day was also considered, as local domestic heating emissions follow distinct diurnal patterns,^30^ offering additional information to differentiate local emissions from transboundary transport. Total OA was chosen to serve as a rough threshold of the model prediction, preventing large deviations from the measured concentrations. In addition, LO-OOA was included as a predictor for MO-OOA due to their common sources during local-emission-dominated periods and the fact that LO-OOA can potentially evolve into MO-OOA through further oxidation.^6,11,14^ Ambient *T* was excluded to avoid potential bias because the training period focused on winter, where *T* is consistently lower compared to summer (Figure S5a). Additionally, local emissions from home heating tend to be higher in winter than during periods in warmer months that have comparable temperatures, while sporadic residential heating during cooler summer periods further complicates the relationship between *T* and local emissions (Figure S5b). RH was excluded since it remains consistently high throughout the year in Ireland (monthly averaged RH > 75%, Figure S5c), providing minimal additional information. WS was not used, because it was already applied in data screening. The selected training data set was randomly split into two parts: 80% for model training and 20% for model testing. The distribution of the number of data points used for model training and testing can be found in Figure S4c. The model was optimized using a grid search, which involves systematically testing a range of hyperparameters and 5-fold cross-validation to ensure reasonable predictions. After evaluation and validation, the model was applied to the entire Dublin data set from 2016 to 2023. Once the local sources-related OOA (LO-OOA_local_ and MO-OOA_local_) were estimated, the contributions from transboundary transport (LO-OOA_TBT_ and MO-OOA_TBT_) were extracted accordingly using equations as below:
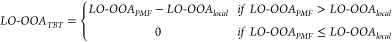

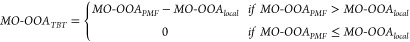


The Monte Carlo simulation^44^ was applied to evaluate the model robustness by randomly omitting 20% of the training data and repeating this process 1000 times. In addition, permutation importance analysis was performed for all predictors to identify critical factors that affect the model’s accuracy, and the partial dependence plots were used to illustrate the relationship between LO-OOA_local_ and MO-OOA_local_ on their predictors. These analyses help identify how different predictors influence the model outputs and provide additional insights into local sources and the underlying processes driving the local formation of an OOA.

## 3 Results and Discussions

### 3.1 OA Identification with Rolling PMF

The six-factor solution was consistently selected as the optimal result for each segmented rolling-PMF analysis over the years. This solution successfully distinguished four POA factors from different fuel types, including Peat, Wood, Coal, and hydrocarbon-like OA (HOA), as well as two OOA factors, i.e., LO-OOA and MO-OOA. Increasing the number of factors did not yield more physically meaningful sources; e.g., as presented in Figure S6, the 8-factor solution resulted in two splitting factors with the r^2^ with LO-OOA reaching as high as 1 for profiles and 0.96 for time series.

**Figure 1:**
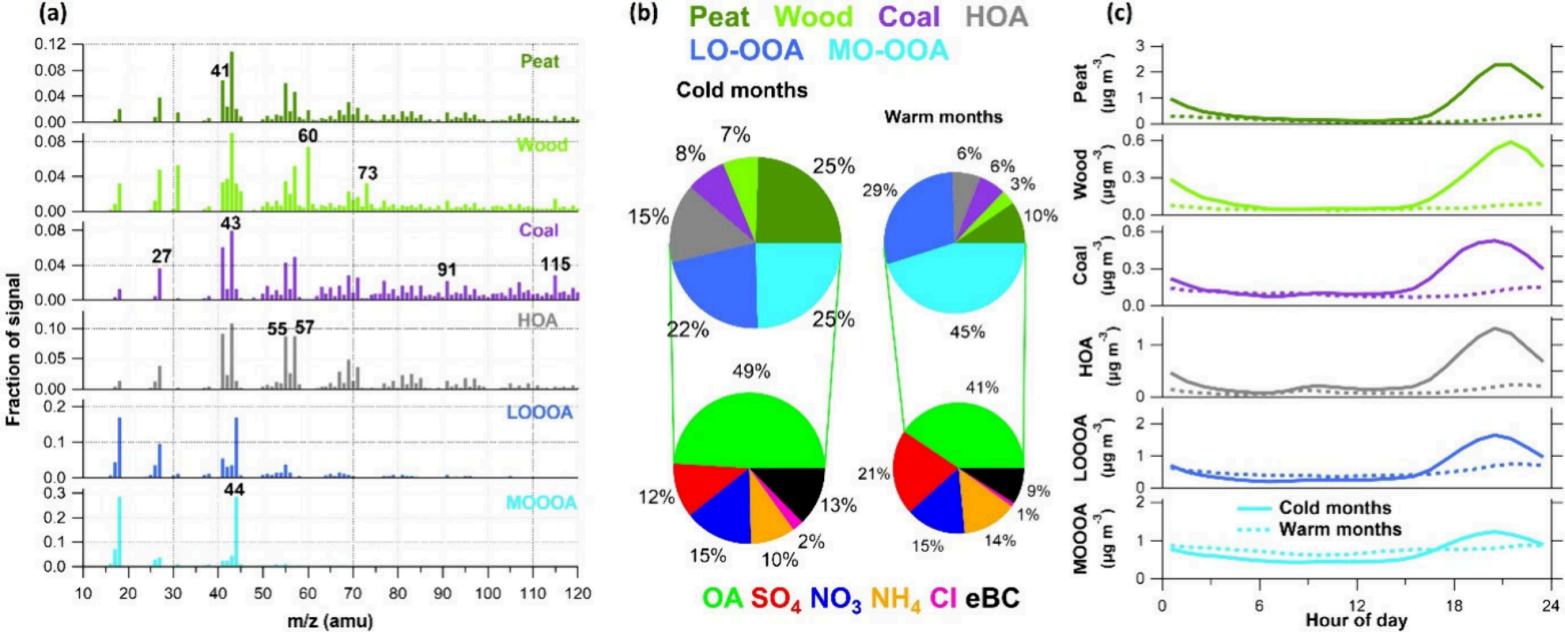
(a) Mass profiles of six OA factors identified by rolling-PMF analysis including Peat, Wood, Coal, HOA, LO-OOA, and MO-OOA, averaged over all segmented analysis periods. (b) The average chemical composition of total PM_1_ and OA during cold (October to March) and warm months (April to September) and (c) diurnal variations of OA factors in cold months (solid lines) and warm months (dashed lines) from 2016 to 2023 in Dublin.

As depicted in Figure 1a, the mass spectra of HOA exhibited apparently stronger signals at *m*/*z*s (mass-to-charge ratios) that are related to hydrocarbon emissions, i.e., C_n_H_2n–1_ (*m*/*z* 27, 41, 55, 69, etc.) and C_n_H_2n+1_ (*m*/*z* 43, 57, 71, etc.), and showed overall high correlation with eBC (r^2^=0.74). It is worth noting that, while HOA is commonly attributed to traffic emissions in many studies,^15,45^ domestic oil burning was identified as a significant source of HOA in Dublin during residential heating hours, characterized by much higher HOA to eBC ratios compared to vehicle emissions.^30^ The mass profile of Wood was characterized by significant contributions at *m*/*z* 60 (0.07) and *m*/*z* 73 (0.03), key tracers of anhydrosugars such as levoglucosan that are produced from the pyrolysis of cellulose during biomass material combustion processes.^46,47^ Coal showed pronounced fractions at m/zs that are tightly correlated with polycyclic aromatic hydrocarbons (PAHs) such as *m*/*z* 91 and 115. Peat had a lower but notable fraction at *m*/*z* 60 (0.02), consistent with the lower cellulose content in peat.^29,39^ These profile signatures serve as useful markers to distinguish POA factors from different sources. Comparatively, the two OOA factors exhibited much higher signal at *m*/*z* 44 (0.19–0.29), which is mostly CO_2_^+^ serving as a good indicator of atmospheric aging,^48,49^ effectively distinguishing them from POA factors (0.01–0.03), with MO-OOA having highest *m*/*z* 44 fraction (0.29). However, both OOA factors are overwhelmingly dominated by *m*/*z*s less than 50 (0.71 for LO-OOA and 0.86 for MO-OOA), providing very limited information to further link them to specific sources. Given that segmented PMF analysis was performed for the Dublin data set, we also evaluated whether the mass profiles of OOA factors significantly changed over time. As displayed in Figure S7, despite minor variations in LO-OOA, both OOA factors exhibited highly consistent mass profiles over the years, with MO-OOA remaining particularly stable (r^2^ = 1.0), indicating the stability of their chemical composition over time.

The pie charts in Figure 1b present the average chemical composition of PM_1_ and OA during different seasons, i.e., cold months (October to March) and warm months (April to September) in Dublin from 2016 to 2023. In the cold months, PM_1_ was dominated by carbonaceous components (OA and eBC), with an average mass contribution of 62%. In addition, POA contributed a significant fraction to total OA at 53%, with Peat being the most significant contributor (25%), followed by HOA (15%), consistent with previous studies that were conducted in Dublin,^30^ indicating significant roles of local domestic heating emissions. Meanwhile, OOA factors (LO-OOA and MO-OOA) also shared a significant portion of total OA in this season (47%). It is important to note that the diurnal variations of both LO-OOA and MO-OOA showed high similarities with POA factors (Figure 1c), with significant elevations during heating hours (17:00–24:00), indicating their common sources. Additionally, OOA factors were often observed to concurrently spike with primary species (POA and eBC) during local events,^29,30^ further suggesting the significant contributions from local domestic heating emissions on OOA. This is likely due to the condensation of semivolatile components and the rapid oxidation of primary species.^29,42^ On the contrary, in warm months, the contributions of secondary inorganic species (SIA, i.e., SO_4_ + NO_3_ + NH_4_) shared equal mass fraction with carbonaceous components. Consistently, OOA contributed more significantly to total OA during warm months (74%), with MO-OOA alone contributing 45% of total OA mass, indicating higher impacts from transboundary transport in this season. In addition, OOA factors showed relatively flat diurnal patterns in warm months, exhibiting typical diurnal variation characteristics of transboundary transport. The results reveal that OOA factors play important roles in causing air pollution events in Dublin, constituting 47–74% of total OA, and are significantly influenced by both local emissions and transboundary transport, with pronounced seasonal variations in composition and sources. However, as noted earlier, traditional PMF analysis was not able to distinguish between local and transboundary OOA.

### 3.2 Evaluation of Model Performance

A supervised SVR ML model was developed to enhance differentiation of the OOA into local and transboundary sources. The high similarity in the PM_1_ and OA composition for selected training data with that of a representative extreme local pollution event (Figure S8a-b) demonstrates the effectiveness of the screening criteria in accurately extracting local emission-dominated pollution events. Additionally, the diurnal patterns of both LO-OOA and MO-OOA mirrored the variations of primary species (Figure S8c), further validating the fundamental assumption of the ML model that OOA was predominantly originated from local sources during local events.

#### 3.2.1 Overall Model Performance

**Figure 2:**
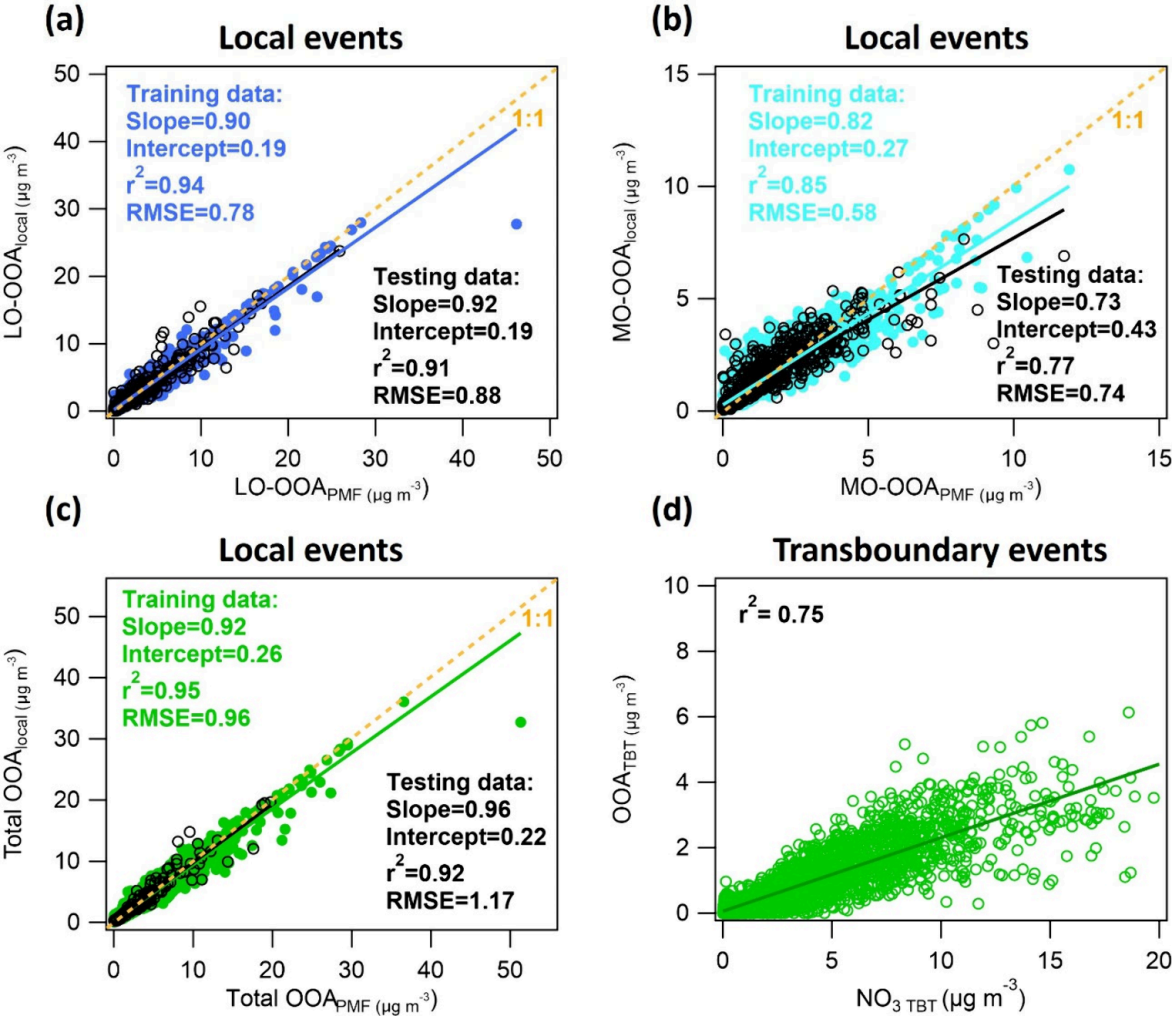
Scatter plots comparing predictions from the model and rolling-PMF analysis for (a) LO-OOA, (b) MO-OOA, and (c) total OOA during selected local events. Panel (d) shows the scatter plot of OOA_TBT_ calculated from the model versus measured NO_3_ filtered for transboundary transport (NO_3 TBT_).

Figure 2 shows the scatter plots of model predicted local OOA versus PMF-derived OOA during selected pollution events dominated by local domestic heating emissions, including comparisons of LO-OOA, MO-OOA, and total OOA. The prediction of LO-OOA_local_ achieved an r^2^ of 0.94 (0.91) for the training (testing) data, which comprises 80% (20%) of the selected data, indicating the ability of the model in effectively capturing the temporary variations of LO-OOA from local sources. The root-mean-square error (RMSE) values, which measure the average magnitude of model prediction errors, were 0.78 μg m^–3^ for training data and 0.88 μg m^–3^ for testing data for LO-OOA, corresponding to 1.7% and 3.4% of the PMF-derived LO-OOA concentrations (0–46 μg m^–3^ for training data and 0–26 μg m^–3^ for testing data, respectively), demonstrating the reliability of the model predictions. A slope close to 1 (0.90–0.92) further validated the strong agreement between the model predictions and PMF-derived LO-OOA during selected local events. Despite higher uncertainty in the prediction of MO-OOA_local_ compared to that of LO-OOA_local_, as shown by the scatter plot in Figure 2b, the model achieved an r^2^ of 0.85 for training data and 0.77 for testing data. Additionally, the RMSE values for MO-OOA were 0.58 μg m^–3^ for training data and 0.74 μg m^–3^ for testing data, being reasonably low at 4.8% and 8.2% of the respective PMF-derived MO-OOA concentrations (0–12 μg m^–3^ for training data and 0–9 μg m^–3^ for testing data respectively). This suggests that the model prediction of MO-OOA_local_ was reasonable. The higher uncertainty in MO-OOA_local_ may arise from the more complex oxidation processes and diverse origins associated with the highly aged nature of MO-OOA,^11,14^ making it more challenging to establish robust relationships with primary species. Even with higher uncertainty in the MO-OOA prediction, the prediction of total OOA achieved overall strong agreement, with r^2^ ranging from 0.92 to 0.95, slopes between 0.92 and 0.96, and low RMSE values of 0.96–1.17 μg m^–3^, corresponding to 1.8–6.5% of the PMF-derived OOA mass concentration. It is worth noting that the ML model predictions tend to be more conservative during periods of extremely high concentrations (OOA > 40 μg m^–3^), leading to slightly underestimated OOA from local sources. This conservative bias can be explained by the rarity of extreme concentrations, which results in less adequate model training in this high concentration range. The box plots in Figure S9a-b illustrate the model’s robustness on a monthly average basis, derived from the Monte Carlo stimulations. The narrow interquartile ranges (25th and 75th percentiles) and short whiskers (10th and 90th percentiles) indicate low variability in the model predictions under various pollution scenarios, especially for LO-OOA_local_. Additionally, the monthly average standard deviation of LO-OOA_local_ and MO-OOA_local_ ranged from 3 to 6% and 7–13% of their respective concentrations, further confirming the strong robustness of the local OOA predictions throughout the year.

Additionally, the model retrieved OOA originating from transboundary sources was compared to NO_3_, a potential tracer of transboundary transport in Ireland.^31,32^ Although its semivolatile nature and contribution of local sources can complicate its temporary variations, NO_3_ remains a valuable indicator of transboundary transport, particularly during episodes with high transboundary contributions when the concentration of NO_3_ can be equal to or even exceed that of OA. A ratio of NO_3_/OA larger than 1.2 was used to filter NO_3_ from transboundary transport (NO_3 TBT_) without significant contributions from local sources. As shown in Figure 2d, the OOA_TBT_ calculated from the model showed a tight correlation with NO_3 TBT_ with r^2^ of 0.75, further indicating that the apportionment of OOA_local_ and OOA_TBT_ is robust and reasonable. This is further supported by their bivariate polar plots. As shown in Figure S10, both LO-OOA_TBT_ and MO-OOA_TBT_ show high concentrations under easterly winds and stagnant conditions, in agreement with previous studies.^31^ In contrast, LO-OOA_local_ and MO-OOA_local_ display distinct local characteristics with no significant dependence on WD, highlighting the effectiveness of the ML model in distinguishing local and transboundary OOA. We also checked instances where OOA_local_ exceeded OOA_PMF_ and found that they mostly (∼90%) occurred at very low concentrations (<0.5 μg m^–3^), where PMF uncertainty in separating OOA factors is highest. Given that this low concentration range is not the primary focus of this study, these overfitting occurrences have a negligible impact on the findings.

The permutation importance analysis assessed the relative influences of all predictors on the model predictions of LO-OOA_local_ and MO-OOA_local_. As depicted in Figure S9c, HOA was found to be the most influential factor on LO-OOA_local_ prediction with a relative importance of 20%, followed by coal (14%), with the relative impacts from all the remaining primary species being significant at 12–13%. While for MO-OOA_local_, the most influential predictor is LO-OOA (17%), which is expected given their common sources during local events and LO-OOA’s potential to form MO-OOA through further oxidation.^6^ The most influential primary species on MO-OOA_local_ are Peat (13%) and Cl (13%). The impacts from WD on both LO-OOA_local_ and MO-OOA_local_ remained insignificant (<1%), further confirming that the Dublin sampling site was not significantly influenced by nearby local point sources.^29^ It is interesting to note that LO-OOA_local_ prediction is slightly more affected by fossil fuel-related POA (e.g., HOA and Coal) than biomass burning-related POA. This may be because of the lower reactivity of key SOA precursors emitted from fossil fuel combustion (e.g., PAHs) under dark conditions,^50^ leading to slower and less efficient oxidation. Additionally, the majority of fossil fuel OA is typically water-insoluble,^51,52^ limiting further aqueous-phase oxidation. These characteristics may result in fossil fuel emissions being less oxidized and, thus, more closely correlated with LO-OOA. The ubiquitous nonlinear relationships between OOA_local_ and primary species (Figure S11) indicated that other factors, such as oxidants availability, may also significantly affect the formation of OOA, which needs further investigation in the future.

#### 3.2.2 Model Performance During Local and Transboundary Pollution Episodes

**Figure 3:**
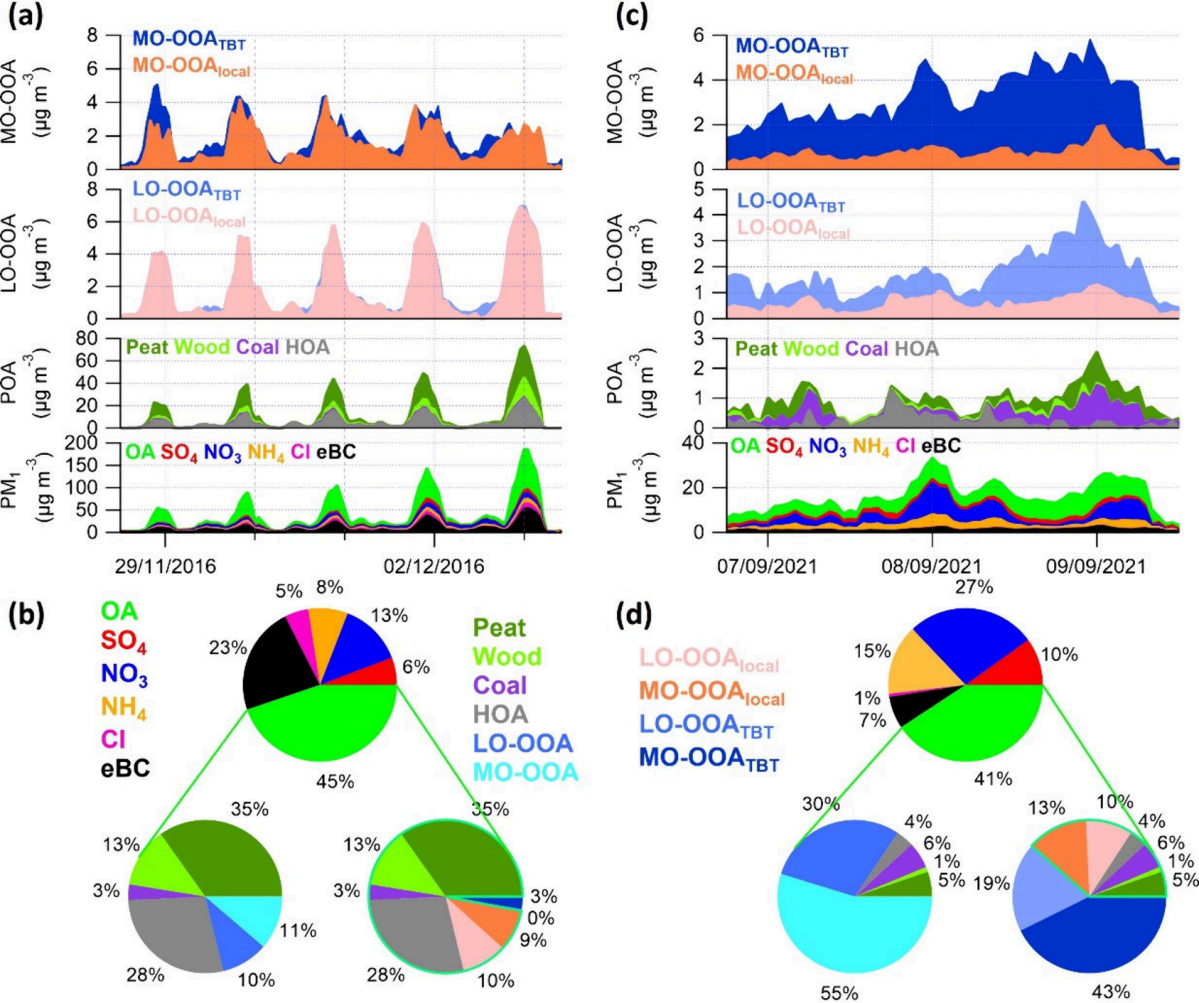
Time series of PM_1_ species including OA, SO_4_, NO_3_, NH_4_, Cl, and eBC, and OA factors including Peat, Wood, Coal, HOA, LO-OOA_local_, LO-OOA_TBT_, MO-OOA_local_, and MO-OOA_TBT_ during selected (a) local- and (c) trans-boundary-dominated pollution episodes. Panel (b) presents the average chemical composition for (a) PM_1_ (top) and OA composition from rolling PMF analysis (bottom left) and the model (bottom right). Panel (d) provides the same data for episode (c).

To further validate the performance of the model, two distinct air pollution events were selected for detailed verification: one dominated by local emissions and one by transboundary sources, neither of which was included in the training data set. As presented in Figure 3a, five consecutive air pollution episodes occurred from November 29th to December 3rd, 2016, with PM_1_ mass concentration peaking from 55 to 186 μg m^–3^. Those pollution events typically started to build up in the late afternoon or early evening (15:00–19:00) when ambient *T* dropped to as low as 0 °C (Figure S12a) and domestic heating started, and gradually dissipated in the early morning hours (00:00 to 05:00). During this period, as shown by the pie charts in Figure 3b, PM_1_ was mainly composed of OA (45%) and eBC (23%). OA was dominated by POA factors (79%), with Peat being the most significant contributor (35%), followed by HOA (28%). Comparatively, secondary species, including SIA and OOA, contributed much less significantly; e.g., SIA accounted for 27% of total PM_1_ mass, and OOA shared 21% of total OA mass. Furthermore, as displayed in Figure 3a, both LO-OOA and MO-OOA spiked concurrently with primary species (POA and eBC), indicating a strong connection with local emissions. This is particularly evident for LO-OOA, which showed a high correlation with POA (r^2^ = 0.97) during this period. The temporary trends and chemical composition clearly highlighted the overwhelmingly dominant role of local emissions.

In agreement with the PMF-derived OOA, which serves as a reference during local-emission-dominated episodes, the model attributed most of the LO-OOA to LO-OOA_local_, especially during the most polluted heating hours, where the model identified more than 98% of LO-OOA as LO-OOA_local_. On average, only a negligible portion (4%) of LO-OOA was attributed to transboundary sources, accounting for less than 0.5% of total OA mass throughout this period. Furthermore, LO-OOA_local_ showed almost no correlation with LO-OOA_TBT_ (r^2^ = 0.09, Figure S13a), confirming that the ML model reliably attributed LO-OOA to its respective local and transboundary sources. For MO-OOA, although a slightly higher fraction (23% on average) was attributed to transboundary sources, the majority of MO-OOA (77%) was linked to local sources, with MO-OOA_local_ typically exceeding 80% of MO-OOA during polluted hours. The lower fraction of MO-OOA_local_ overall could be due to the ubiquitous background and higher model uncertainty caused by its more complex atmospheric processes. Overall, the model showed strong agreement with PMF-derived OOA during air pollution episodes dominated by local domestic heating emissions.

Figure 3c presents an air pollution episode dominated by transboundary transport. During this event, PM_1_ started to increase from the evening of September 6th, 2021, from around 6 μg m^–3^ to above 30 μg m^–3^ under easterly winds (Figure S12b), lasting until the noon of September 9th with stagnant meteorological conditions (WS < 5 m s^–1^). NO_3_ and MO-OOA showed the most significant increases, rising from around 0.5 μg m^–3^ to over 14 μg m^–3^ and 1.5 μg m^–3^ to around 6 μg m^–3^, respectively. On average, PM_1_ was predominantly composed of SIA (Figure 3d), with NO_3_ being the largest contributor (27%), followed by NH_4_ (15%). Although OA remained the most significant component of PM_1_ (41%), the contribution from eBC was minor, at 7%. Importantly, OA was overwhelmingly composed of OOA, with MO-OOA alone accounting for 55% of total OA mass, and LO-OOA also contributing significantly at 30%. In contrast, POA factors showed minor contributions, with their average mass fractions ranging from 1 to 6% and mass concentration under 1 μg m^–3^, showing no significant increase during typical heating hours. The significant contributions of NO_3_ and MO-OOA, alongside the much smaller fractions of primary species, clearly highlighted the dominant role of transboundary sources. Consistently, the model attributed 65% of LO-OOA and 77% of MO-OOA to transboundary transport, aligning well with the transboundary characteristics of this pollution episode. It is worth noting that, although local emissions contributed much less significantly than transboundary sources during this episode, POA factors still accounted for an average of 16% of total OA mass, peaking at 30% during rush hours when traffic emissions were high. This indicates that local sources may still play a notable role in OOA formation, especially for LO-OOA which is fresher and more strongly influenced by local emissions.^53^ Both LO-OOA_local_ and MO-OOA_local_ showed tight correlations with POA, with r^2^ values of 0.71 and 0.68, respectively, suggesting that the model is able to effectively capture OOA from local sources even under significant impacts of transboundary transport.

#### 3.2.3 Model Application on Mixed Pollution Episode

**Figure 4:**
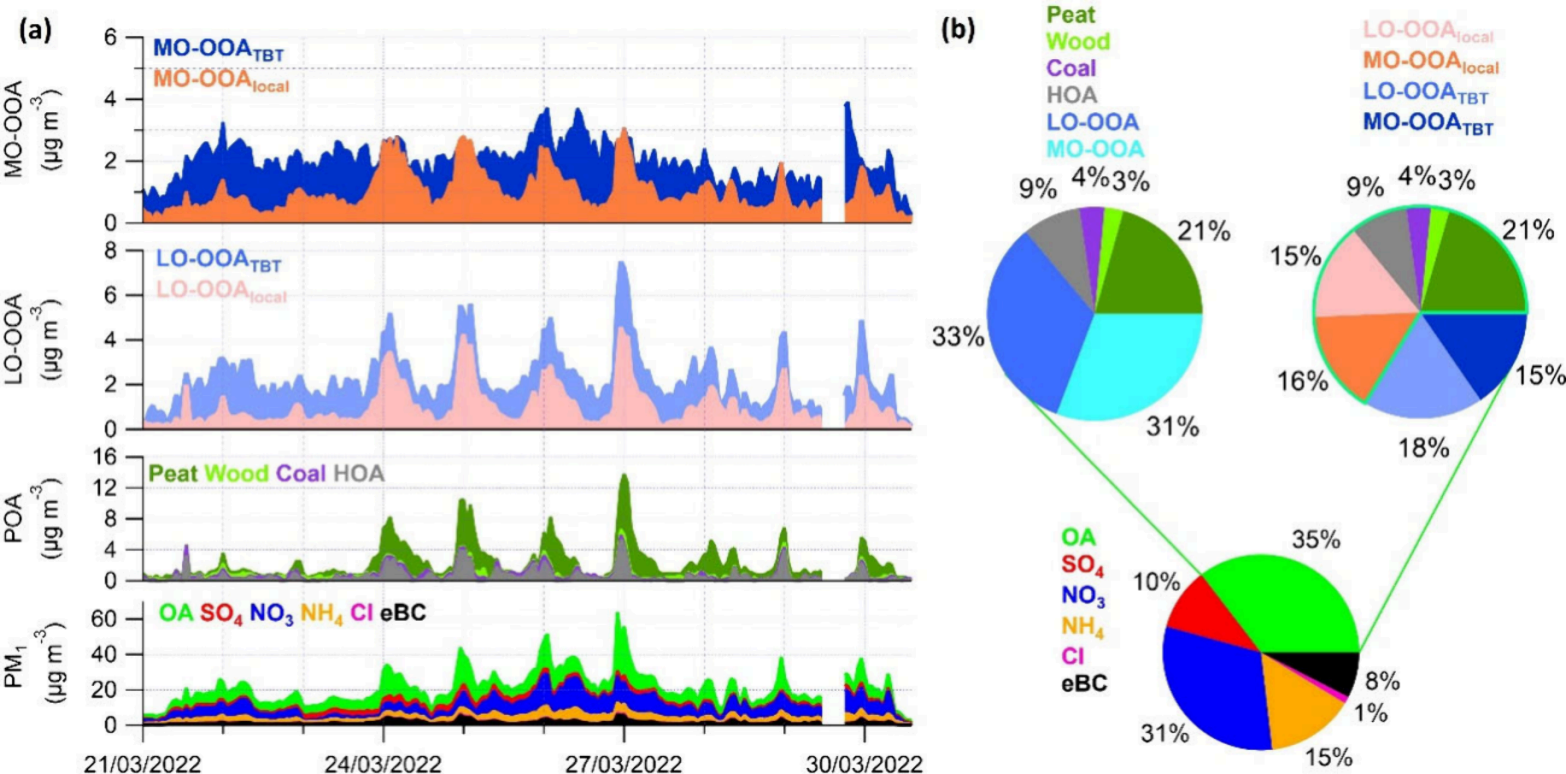
(a) Time series of PM_1_ species including OA, SO_4_, NO_3_, NH_4_, Cl, and eBC, and OA factors including Peat, Wood, Coal, HOA, LO-OOA_local_, LO-OOA_TBT_, MO-OOA_local_, and MO-OOA_TBT_ during selected mixed pollution episode in March 2022. Panel (b) shows the average chemical composition of PM_1_ (bottom) and OA from rolling PMF analysis (top left) and model (top right) during this episode.

Mixed air pollution events, where both transboundary transport and local emissions play substantial roles, pose significant challenges for traditional PMF to attribute OOA from different origins. As shown in Figure 4a, an air pollution event started to build up from the evening of March 21st, 2022, with the mass concentrations of NO_3_ and OOA significantly increased from <2 μg m^–3^ to ∼19 μg m^–3^ and ∼10 μg m^–3^, respectively. Favored by stagnant meteorological conditions (e.g., WS < 5 m s^–1^, Figure S14), this pollution episode lasted a few days until the morning of March 30th, 2022. The significant enhancement of NO_3_ and OOA, associated with the relatively long duration of this pollution episode, indicate strong impacts from transboundary transport.^31^ Meanwhile, significant contributions from local domestic heating emissions were observed at night, especially from March 24th to 30th when *T* dropped below 5 °C. For example, during this period, the mass concentrations of POA and eBC spiked to 8–14 μg m^–3^ and 4–6 μg m^–3^ respectively at night, while their daytime mass concentrations remained below 2 μg m^–3^. Overall, as presented in Figure 4b, OA was the most dominant species during this episode, with the average mass fraction of 35%, accompanied by a notable contribution of eBC (8%). Although the contribution from all POA was minor compared to typical local events (36% vs >50%),^30^ the fraction can reach up to 60% at night, indicating significant influences from local domestic heating. On the other hand, the fractional contribution of NO_3_ to total PM_1_ was nearly equal to OA at 31%, and the OOA factors dominated total OA with fractions of LO-OOA and MO-OOA being close at 33% and 31%, respectively. The results reveal that both local residential heating emissions and transboundary transport contributed significantly to this episode, but a clear breakdown of their relative contributions could not be achieved using PMF analysis alone.

The temporal variations of LO-OOA_local_ and MO-OOA_local_ predicted by the ML model showed clear local emission characteristics (Figure 4a), with significant concentration increases only at night, spiking to 4.6 μg m^–3^ and 3.0 μg m^–3^, respectively. These increases coincided with typical residential heating hours, which were highly consistent with POA and eBC. The correlation coefficients with POA reached 0.96 for LO-OOA_local_ and 0.80 for MO-OOA_local_, respectively. Comparatively, the r^2^ values of PMF-derived LO-OOA and MO-OOA with POA were 0.78 and 0.02, respectively (Figure S15a-b). LO-OOA_TBT_ and NO_3_ displayed similar temporal trends, with both showing notable enhancements at night, likely due to their semivolatile properties.^11,54^ Impacts from the shallower nocturnal boundary layer are also expected, and local residential heating emissions may also have played a role in the NO_3_ increase. However, it is important to acknowledge that the nighttime enhancement of LO-OOA_TBT_ may be linked to the possible underprediction of LO-OOA_local_, as the most pronounced LO-OOA_TBT_ increase coincided with the strongest local emissions, despite their overall weak correlation (r^2^ = 0.20, Figure S13d). MO-OOA_TBT_ showed a strong correlation with NO_3 TBT_ during daytime (r^2^ = 0.79, Figure S15c) when residential heating emissions were minimal (POA < 1.5 μg m^–3^), while this correlation weakened at night (r^2^ = 0.57). The weaker correlation may result from their different volatilities; however, the MO-OOA_TBT_ concentration showed notable decreases during nights when local domestic heating emissions were particularly strong (March 24th to 29th), implying that MO-OOA_local_ might have been slightly overpredicted during these nights. Despite some uncertainties, the results elucidate that the model is capable of providing reasonable quantifications of local and transboundary sources, even during mixed pollution episode.

Although both local and transboundary sources contributed significantly during this mixed pollution episode, their relative importance varied by time of day. During nonheating hours (POA < 1.5 μg m^–3^), NO_3_ contributed slightly more to total PM_1_ than OA (33% vs 31%, Figure S16a), and OOA_TBT_ dominated OA with the average fraction at 56%. This qualitatively aligns with the traditional PMF analysis, where OOA constituted 80% of total OA, indicating the dominant role of transboundary transport. In contrast, during heating hours, although NO_3_ still contributed significantly at 30% (Figure S16b), the contribution of OA increased to 38%, associated with a notable rise of eBC (6% vs 9%). PMF analysis suggested that OOA still dominated OA at 55%, although POA fractions significantly increased from 20% to 45%. However, the quantitative results from the model elucidated that more than half (61%) of the OOA originated from local emissions. On average, OA from local sources (POA, LO-OOA_local_, and MO-OOA_local_) accounted for 78% of total OA, highlighting the more critical role of local emissions during heating hours. Over the span of this mixed pollution episode, nearly half of both LO-OOA (45%) and MO-OOA (50%) were emitted from local sources, and the remaining contributions came from transboundary transport (Figure 4b). Over the mixed pollution episode, OA originated from local sources contributed 68% of total OA despite the strong influences from transboundary sources, indicating that local emissions played more critical roles, especially at night. However, since the traditional PMF analysis showed dominance of OOA (64%), the lack of detailed OOA origin information could lead to contrary conclusions about transboundary sources, ultimately resulting in ineffective control strategies.

## 4 Conclusions

This study demonstrated the capability and effectiveness of a machine learning model in enhancing the source attribution of OOA for the Dublin data set from 2016 to 2023. While the rolling-PMF analysis revealed that the OOA accounted for a significant fraction of total OA in Dublin (47–74%), it lacked the ability to differentiate the OOA from different sources. In contrast, the machine learning model successfully distinguished OOA contributions from local and transboundary sources, providing quantitative insights into their relative impacts. The model exhibited strong agreements with PMF-derived OOA during local emission-dominated events, with robust predictions reflected by low RMSE values and further supported by Monte Carlo simulations. During events dominated by local emissions, the model successfully attributed most of the LO-OOA (96%) and MO-OOA (77%) to local sources. Similarly, during pollution episodes dominated by transboundary transport, the model effectively attributed the majority of the OOA (66–77%) to transboundary sources. The relative importance analysis indicated that LO-OOA_local_ prediction was more affected by fossil fuel emissions, such as HOA (20%) and coal (14%), while MO-OOA_local_ was primarily influenced by LO-OOA (17%), implying their potential sources and formation mechanisms. By applying the model to a mixed pollution episode, the findings underscored that despite substantial contributions from transboundary transport during this episode, local emissions from residential heating were more critical sources of OA, with local OA on average accounting for 68% of total OA and reaching 78% during heating hours. This highlights the continued need to reduce local OA emissions and the importance of distinguishing local OOA sources from transboundary transport for effective air pollution control strategies. The successful application of the machine learning model in this study demonstrated the significant advantages of machine learning in enhancing the OOA source apportionment, with potential for broader applications beyond the OOA. However, this machine learning method also has certain limitations. While it effectively differentiates OOA from local and transboundary sources, incorporating oxidants data (e.g., O_3_ and NOx) could further reduce model uncertainty, especially for MO-OOA. The approach currently relies on observational and statistical methods, but validation against modeling results could further strengthen confidence in its applicability. In addition, although trained on a valuable multiyear data set (2016–2023), regular updates with new ambient data are needed to account for variability in emission sources over time.

## Supporting Information Available

Details of NR-PM1 measurements in Dublin (S1.1), details of the rolling PMF analysis (S1.2), ML model evaluation and optimization (S1.3), detailed meteorological conditions (Figures S5, S12, and S14), 8-factor rolling PMF results (Figure S6), comparisons of the training data set with local events (Figure S8), uncertainty analyses for model predictions (Figure S9a-b) and relative importance analysis of predictors (Figure S9c-d), analysis of partial dependences of predictors (Figure S11), correlation plots for OOA factors and other key species (Figure S15), and average OA and PM_1_ composition during different hours (Figure S16) (PDF)
