# Supplementary material for: Enhancing Differentiation of Oxygenated Organic Aerosol: A Machine Learning Approach to Distinguish Local and Transboundary Pollution
Source: ACS EST Air. 2025 Apr 15;2(5):891–902. doi: 10.1021/acsestair.4c00331 (PMC12070415; doi:10.1021/acsestair.4c00331)
Supplement: Supplementary file 1 — ea4c00331_si_001.pdf [file ea4c00331_si_001.pdf]

Supporting information of

Enhancing Differentiation of Oxygenated Organic Aerosol: A Machine Learning Approach to Distinguish Local and Transboundary Pollution

Lu Lei[1], Wei Xu[2], Chunshui Lin[3], Baihua Chen[2], Kirsten N. Fossum[1], Darius Ceburnis[1], Colin O'Dowd[1] and Jurgita Ovadnevaite[1*]*

[1]School of Natural Sciences, Ryan Institute's Centre for Climate & Air Pollution Studies, University of Galway, Galway, H91 CF50, Ireland.

[2]Center for Excellence in Regional Atmospheric Environment, Institute of Urban Environment, Chinese Academy of Sciences, Xiamen, 361021, China.

[3]State Key Laboratory of Loess and Quaternary Geology and Key Laboratory of Aerosol Chemistry and Physics, Institute of Earth Environment, Chinese Academy of Sciences, Xi'an, 710061, China.

KEYWORDS: Oxygenated organic aerosol, Source apportionment, Rolling PMF, Machine learning, Urban air pollution.

S1 Supplementary Discussion

S1.1 Instrumentation for NR-PM_1 measurements in Dublin (2016-2023)

Non-refractory PM_1 (NR-PM_1) species were measured using a quadrupole aerosol chemical monitor (Q-ACSM) at the Dublin urban background site from 2016 to 2023. The same Q-ACSM (ACSM1) was deployed throughout this period, except for two short periods from 2018 may to 2018 July (ACSM3) and 2018 September to 2019 March (ACSM2) when two other ACSMs were deployed (Table S1). These temporary replacements were necessary to maintain continuous measurements while ACSM1 was unavailable. All ACSMs deployed at the Dublin site were regularly calibrated following the standard calibration procedures [1]. To assess instrument stability, the response factor (RF) of ACSM1 was analyzed over time, with its variations presented in Figure S1a. As shown by the box plot, ACSM1 showed little variations in RF (CV < 10%), with an average of 3.0e-11 and standard deviation of 2.7e-12, suggesting the long-term stability of the instrument. To ensure data consistency across the different ACSMs, measurements from ACSM2 and ACSM3 were intercompared with ACSM1 to confirm good agreement. All datasets were processed using standard data analysis software. Validation of the entire dataset were performed by comparing with collocated SMPS and $PM_{2.5}$ data from a nearby EPA monitoring station. These intercomparisons, which were conducted on hourly resolution data, confirmed the reliability of the long-term dataset used in this study (Figure S1b-c). The detailed instrumentation information and data coverage of hourly averaged measurements in each year are summarized in Table S1, and the concentration ranges of PM_1 species for each year are presented in Table S2.

Table S1. Summary of the sampling duration, instruments used, hourly data coverage, and number of data points for the Dublin NR-PM_1 dataset from 2016 to 2023.

Year	Duration	Instrumentation	QA Data coverage	Number of data points
2016	05/08 – 31/12	ACSM1	41%	3482
2017	01/01 – 31/12	ACSM1	96%	8426
2018	01/01 – 15/05 & 16/05- 26/07 & 05/09 – 31/12	ACSM1 & ACSM3 & ACSM2	87%	7596
2019	01/01 – 12/03 & 18/09 – 31/12	ACSM2 & ACSM1	45%	3925
2020	01/01 – 31/12	ACSM1	92%	8096
2021	01/01 – 31/12	ACSM1	98%	8570
2022	01/01 – 31/12	ACSM1	92%	8083
2023	01/01 – 31/12	ACSM1	95%	8346

Table S2. Summary of the mass concentration ranges of NR-PM_1 species in Dublin for each year from 2016 to 2023.

Year	OA ($\mu g\ m^{-3}$) avg ± sdv (max)	SO_4 ($\mu g\ m^{-3}$) avg ± sdv(max)	NO_3 ($\mu g\ m^{-3}$) avg ± sdv (max)	NH_4 ($\mu g\ m^{-3}$) avg ± sdv (max)	Cl ($\mu g\ m^{-3}$) avg ± sdv (max)	eBC ($\mu g\ m^{-3}$) avg ± sdv (max)
2016	3.1±9.4 (222.2)	0.9±1.2 (23.2)	1.1±1.9 (16.2)	0.9±1.2 (16.4)	0.2±0.8 (18.6)	1.1±3.2 (53.2)
2017	1.8±4.9 (147.3)	0.8±0.9 (17.9)	0.9±2.0 (28.2)	0.7±0.0 (15.3)	0.1±0.4 (13.2)	0.6±1.9 (70.7)
2018	3.0±8.6 (271.8)	1.0±1.1 (19.8)	1.0±1.8 (17.7)	0.7±1.0 (13.4)	0.1±0.5 (21.0)	0.7±2.1 (65.2)
2019	4.3±9.0 (156.3)	0.9±1.0 (8.3)	1.0±2.1 (22.1)	0.7±1.1 (10.5)	0.1±0.3 (5.0)	1.0±2.2 (37.5)
2020	2.7±7.1 (222.6)	0.7±0.9 (17.2)	0.7±1.5 (16.9)	0.5±0.8 (10.3)	0.1±0.3 (9.9)	0.6±1.0 (54.4)
2021	2.7±4.8 (92.4)	0.9±0.8 (7.5)	0.9±1.6 (15.8)	0.7±0.9 (7.6)	0.1±0.2 (3.7)	0.5±0.9 (19.3)
2022	2.2±3.7 (67.8)	0.9±1.0 (11.6)	0.7±1.5 (18.3)	0.5±0.8 (6.8)	0.1±0.2 (6.3)	0.5±0.9 (18.8)
2023	1.9±3.7 (78.2)	0.7±0.8 (12.4)	0.5±0.9 (10.4)	0.4±0.6 (5.1)	0.1±0.2 (6.0)	0.5±1.0 (17.5)

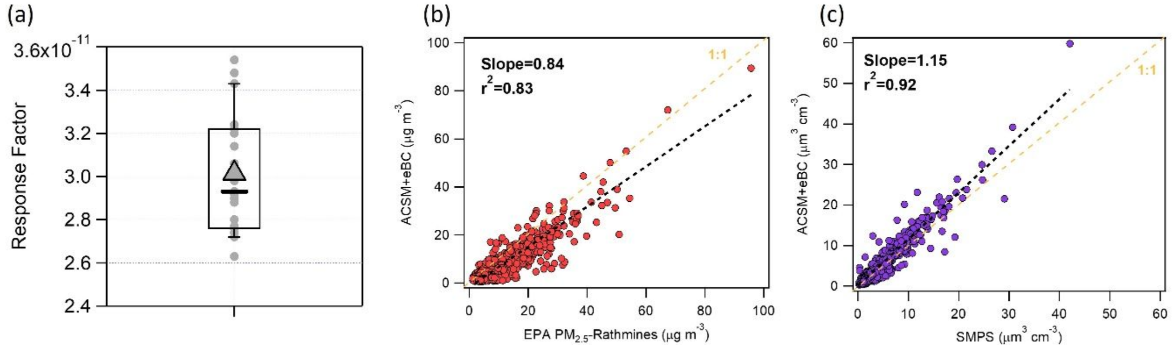

Figure S1. (a) Box plot showing the variations in the response factor (RF) of ACSM1 from 2016 to 2023. The mean (triangle), median (horizontal line), 25th and 75th percentiles (box), and 10th and 90th percentiles (lower and upper whiskers) of RF over the 21 calibrations are shown. Scatter plots showing the intercomparisons of the multi-year PM_1 measurements (Q-

ACSM+AE33, i.e., NR-PM_1+eBC) in Dublin with (b) $PM_{2.5}$ measurements at Rathmines monitor station and (c) volume concentrations measured by collocated SMPS system.

S1.2 OA Source Apportionment

Positive Matrix Factorization (PMF) with the Multilinear Engine (ME-2) was applied to attribute the measured organic aerosol (OA) into different sources using SoFi pro (version 9.4 [2]) by constraining POA factors. The PMF model is expressed as:

$$\mathbf{X} = \mathbf{GF} + \mathbf{E}$$

where X is the of time-series matrix of organic ions, approximated by G (factor time series) and F (mass spectral profile), with E representing model residuals [3]. Although PMF required non-negative outputs, it faces rotational ambiguity, where multiple G and F combinations yield similar residuals but not all represent physically meaningful solutions. The ME-2 algorithm mitigates this issue by incorporating prior information such as known factor profiles, guiding PMF analysis toward environmentally realistic solutions.

In this study, POA factor profiles for peat, wood and coal were constrained based on previous burning experiment [4], while hydrocarbon-like OA (HOA) was anchored using a reference profile from urban Paris [5]. To allow flexibility, the a-value approach was used, enabling constrained factors to vary within a defined range from their reference profiles. For example, an a-value of 0.2 permits 20% variation, while a-value of 1 corresponds to completely unconstraint free PMF. Furthermore, instead of applying an uniform a-value for all ions, the “limits” a-value approach was used, allowing each individual m/z to vary independently as needed [4].

Traditional PMF assumes static OA factor profiles throughout an entire campaign or season, which can introduce uncertainties due to the dynamic nature of OA. To address this, rolling PMF with a 14-day moving window and daily shifts was implemented in SoFi Pro [6]. This time-dependent source apportionment strategy, combined with bootstrap resampling, allowed for factors profiles to adapt continuously to changing atmospheric conditions and sources.

We applied the same constraining strategies and bootstrap resampling used in our previous studies [4, 7]. In brief, a bootstrap-based resampling strategy with a total of 100 runs was applied to examine the statistical uncertainty. The bootstrap approach generates a new input matrix by randomly resampling of rows from the original matrix. Resulting PMF runs were averaged to obtain the optimized solution, with the standard deviation reflecting the model uncertainty. A set of predefined criteria was used to select solid PMF runs, as summarized in Table S3. Specifically, HOA was evaluated based on its strong correlations with external eBC during morning rush hours (06:00-10:00, p value $\leq$ 0.05), while the total POA (peat + wood + coal + HOA) were assessed based on their correlation with eBC during evening heating hours (18:00-23:00, p value $\leq$ 0.05). The explained variation in m/z 60 by wood and peat was also considered a crucial criterion for biomass burning representation. To distinguish between OOA factors, the fraction of m/z 44 and m/z 43 in the mass spectra was examined, ensuring that OOA factors (Factor_5 and Factor_6 as free factors) contained non-zero contributions of these key markers for OOA. Only PMF runs that met these criteria were averaged to obtain the final solution, with uncertainties represented as one standard deviation. Additionally, the fraction of m/z 44, whose presence suggests extensive atmospheric processing and aging, in total organic ions (f_{44}) was used to differentiate more oxidized OOA (MO-OOA) from less oxidized OOA (LO-OOA).

To further account for potential source variabilities over time, the PMF analysis was not performed on the entire dataset from 2016 to 2023 as a single unified analysis. Instead, in addition to periods when two other ACSMs were deployed, the dataset from ACSM1 was divided into multiple periods: (1) August 2016 to May 2018, (2) September 2019 to December 2020, and (3) followed by separate yearly PMF analyses for 2021-2023. This segmentation allowed OA profiles to change dynamically. However, to maintain consistency, the same criteria and optimization approaches were applied across all periods. The residuals for each segmented rolling PMF analysis were evaluated and found to be well-balanced, showing no systematic over- or underestimation.

Table S3. Criteria for selecting PMF runs in the rolling PMF analysis used in this study.

Criterion	Type	Threshold
HOA vs eBC	r^2, time series during the morning rush hours (6:00-10:00)	$p \leq 0.05$
HOA+peat+wood+coal vs eBC	r^2, time series during the evening heating hours (18:00-23:00)	$p \leq 0.05$
Explained variation in m/z 60 by wood+peat	Average, normal time series	$p \leq 0.05$
Factor_5 [44]	Profiles, fraction, sorting criterion	> 0
Factor_5 [43]	Profiles, fraction	> 0
Factor_6 [44]	Profiles, fraction	> 0
Factor_6 [43]	Profiles, fraction	> 0

S1.3 SVR Model Performance Evaluation and Optimization

The selected criteria for filtering training data, and the predictors used for the ML model are summarized in Table S4. The choice of criteria thresholds in this study aimed to strike a balance between isolating the "purest" local events and ensuring a sufficient number of data points for reliable model training. To better assess the sensitivity of the five selected training data filtering criteria, we performed a stratified data perturbation analysis specifically for MO-OOA as an

example. This approach systematically evaluates the robustness of the ML model by introducing controlled amounts of data that violate individual selection criteria into the original local event dataset. More specifically, we first established a baseline SVR model on the original dataset, which was filtered using the 5 selected criteria. The model's performance on a held-out test set (evaluated via RMSE and r^2) served as the reference (0%). Next, we perturbed the dataset by selectively adding data that violated each criterion, generating modified datasets with contamination levels ranging from 10% to 50%. Specifically, we added data that: (1) had WS > 5 m s^{-1}, (2) showed OA concentration < 0.5 μg m^{-3}, (3) was outside of winter months (April to September), (4) had a POA fraction lower than 50% and (5) had an $OA/NO_3 < 2$. We then retrained the ML model on each perturbed dataset and evaluated its performance using the same test set. The results, as shown in Figure S3a, demonstrate that the model performance remained largely robust to variations in individual criterion thresholds. No single threshold significantly impacts the model's performance, as RMSE remains within between 0.74 to 0.76 μg m^{-3}, except for higher perturbations caused by OA/NO_3 ratio (RMSE=0.74-0.78 μg m^{-3}). A lower OA/NO_3 ratio (< 2) may introduce periods influenced by transboundary sources, which explains the higher sensitivity to this criterion. The five selected criteria were chosen because they are all strongly correlated with local heating emissions, which may explain why the model's performance remains stable despite violations of individual thresholds. This robustness indicates that the model effectively captures key patterns in local emissions without being overly dependent on specific thresholds. By combining these five criteria, we ensure effective filtering of local emission pollution episodes while also maintaining a sufficient number of data points for reliable model training.

The predictive variables used as inputs for the ML model were selected through optimization to achieve the best model performance. For instance, as shown in Figure S4a-b, excluding WS reduced overestimation at lower concentrations by preventing bias from the training dataset, which included only periods with WS < 5 m s^{-1}. Similarly, incorporating eBC and HOA helped reduce the model's conservative bias during high-concentration periods, as these variables effectively capture primary combustion influences on local OOA. For another example, we also tested the model performance with and without ambient T and RH as predictive variables. The results showed no significant improvement, with r^2 between MO-OOA_{local} and MO-OOA_{PMF} remaining nearly unchanged (within 0.02). Similar optimization was also applied to the other predictors.

In addition to the theoretical advantages of SVR, we also compared its model performance with those from other machine learning models, including Random Forests (RF), eXtreme Gradient Boosting (XGB), and Deep Neural Networks (NN). As shown in Figure S3b, model performance on the training dataset was evaluated using root mean square error (RMSE). While the performance of the various machine learning models was comparable, SVR demonstrated a slightly lower RMSE with a narrower standard deviation, indicating its greater suitability for OOA differentiation. However, it's important to note that the primary focus of this paper is not to assess the impact of specific machine learning algorithms, which is why we did not explore further details beyond this.

Table S4. Summary of the filtering criteria for the training dataset and the selected predictors of the ML model. A check mark (√) indicates a variable was used as a filtering criterion or model predictor, while (×) indicates it was not used.

Variables	Criteria	Predictor
OA	√ (OA>0.5 μg m^{-3})	√
SO_4	×	×
NO_3	×	×
OA/NO_3	√ (OA/NO_3>2)	×
NH_4	×	×
Cl	×	√
eBC	×	√
Peat	×	√
Wood	×	√
Coal	×	√
HOA	×	√
POA fraction	√ (POA fraction > 50%)	×
LO-OOA	×	√ (for MO-OOA only)
MO-OOA	×	×
WS	√ (WS < 5 m s^{-1})	×
WD	×	√
Temp	×	×
RH	×	×
Hour of day	×	√
Month of year	√ (October-March)	×

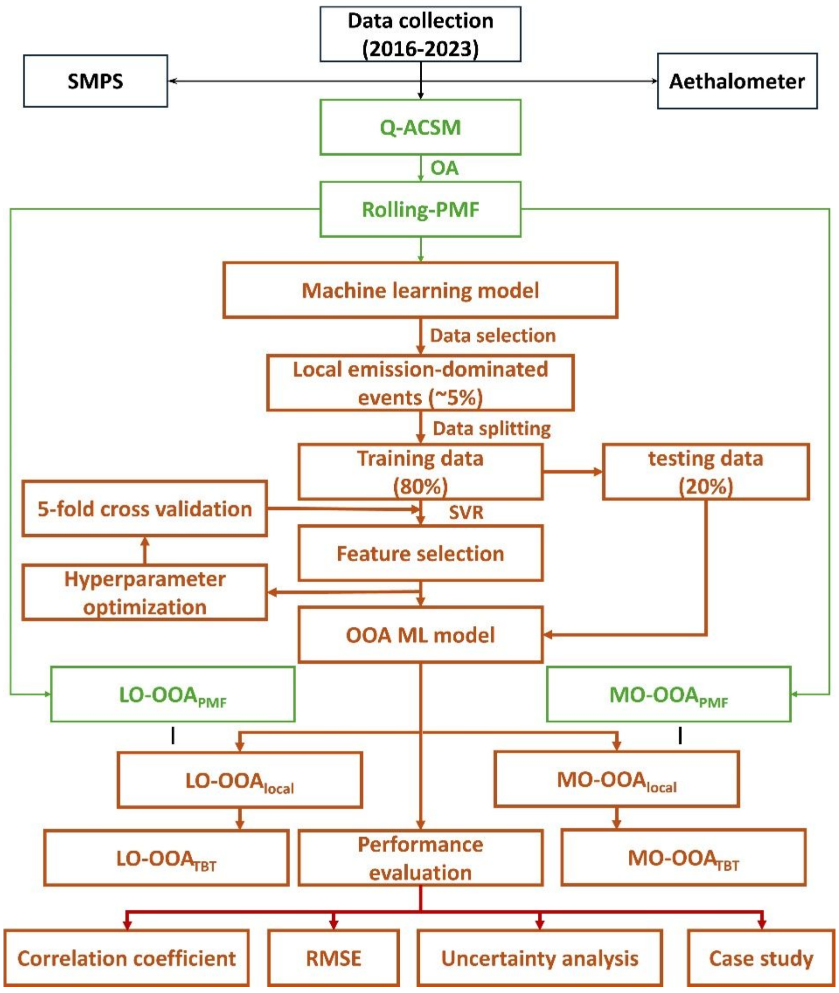

Figure S2. Schematic plot showing the development of the ML model in this study.

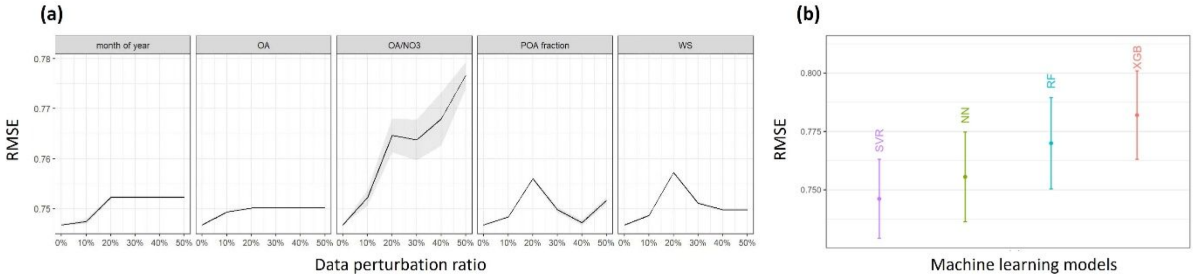

Figure S3. (a) Variations in the ML model RMSE of MO-OOA under data perturbation ranging from 10% to 50%, applied to all five filtering criteria to evaluate model sensitivity. (b) Model performance of different machine learning algorithms evaluated using RMSE, with error bars representing the standard deviation.

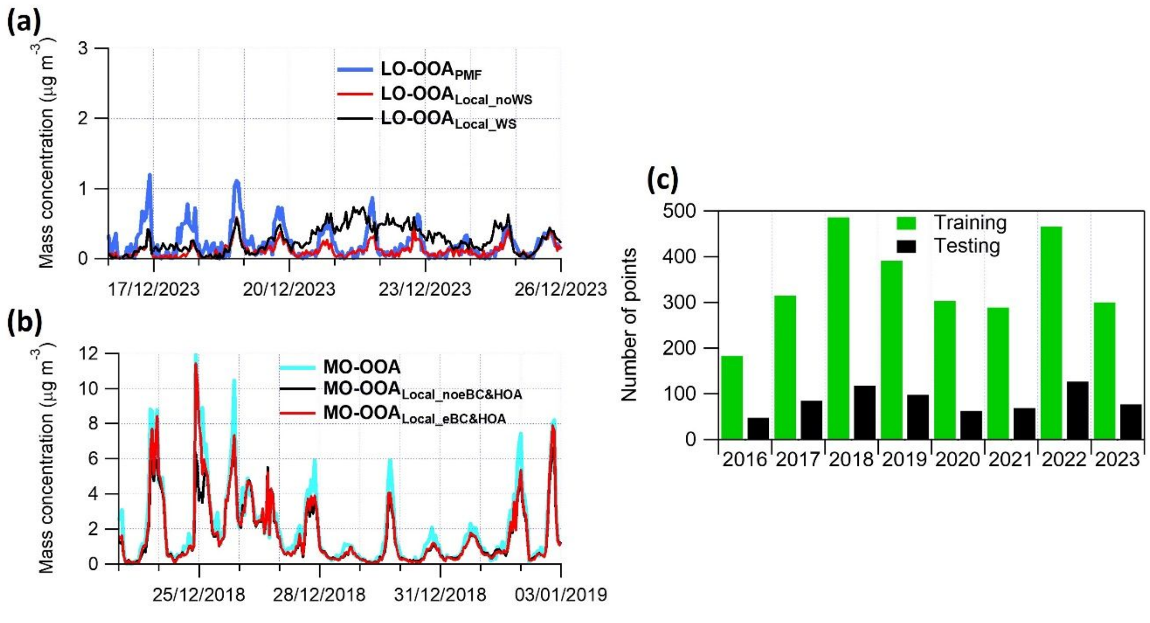

Figure S4. Examples showing the optimization of model predictor selection, demonstrating the effects of (a) WS and (b) eBC and HOA on model performance. (c) shows the distribution of data points used for model training and testing each year from 2016 to 2023.

S2 Supplementary Figures

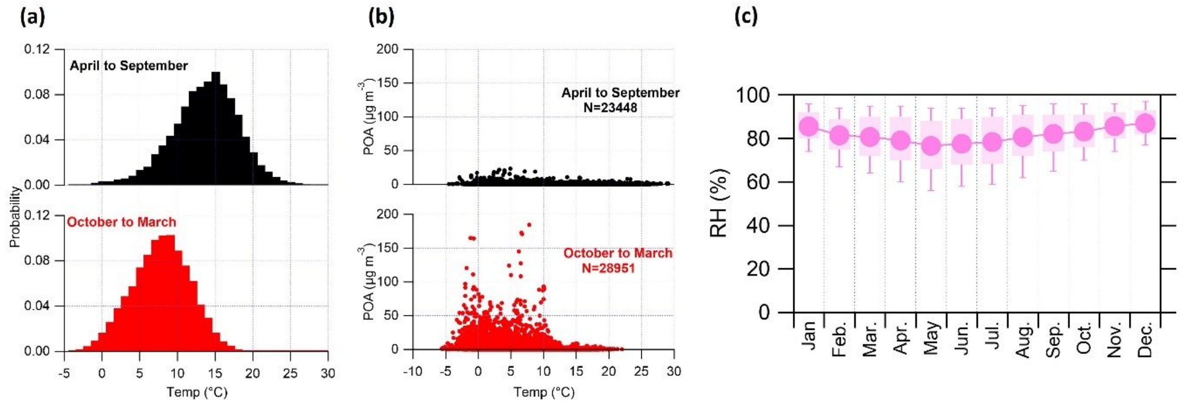

Figure S5. (a) The probability distribution of ambient T in cold months (October to March) and warm months (April to September), (b) the scatter plots of ambient T and POA mass concentrations during cold and warm months. Panel (c) shows the monthly distribution of RH in Dublin from 2016 to 2023. The mean (dots), median (horizontal lines), 25th and 75th percentiles (lower and upper boxes), and 10th and 90th percentiles (lower and upper whiskers) of RH in each month are shown.

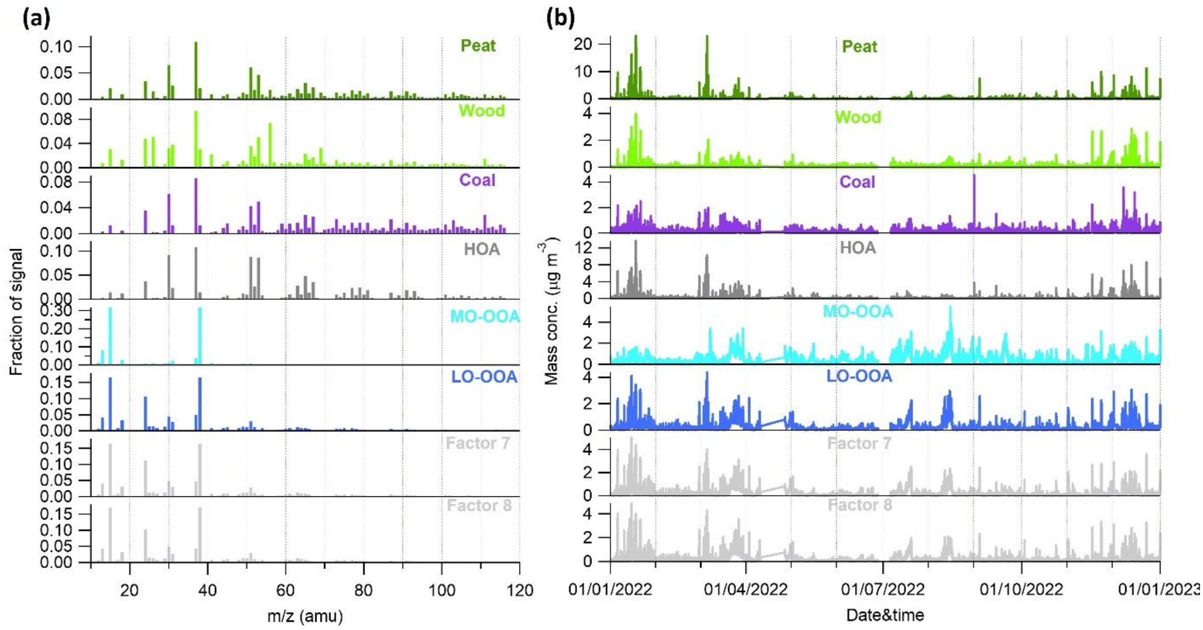

Figure S6. (a) Mass profiles and (b) time series of OA factored identified by 8-factor rolling PMF solution, including Peat, Wood, Coal, HOA, LO-OOA, MO-OOA and two splitting factors (factor7 and 8).

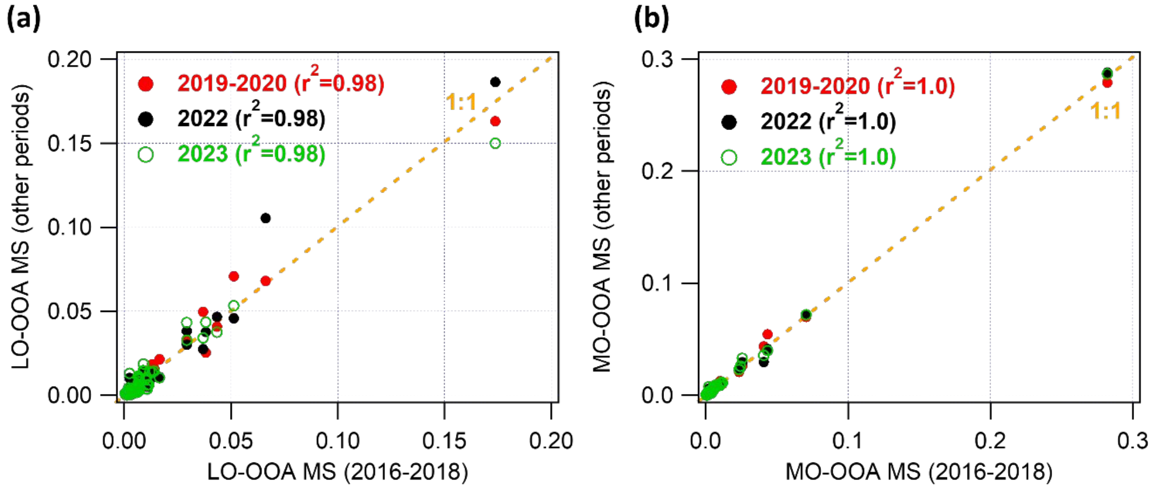

Figure S7. Scatter plots comparing the mass profiles of (a) LO-OOA and (b) MO-OOA from different years to those from 2016–2018.

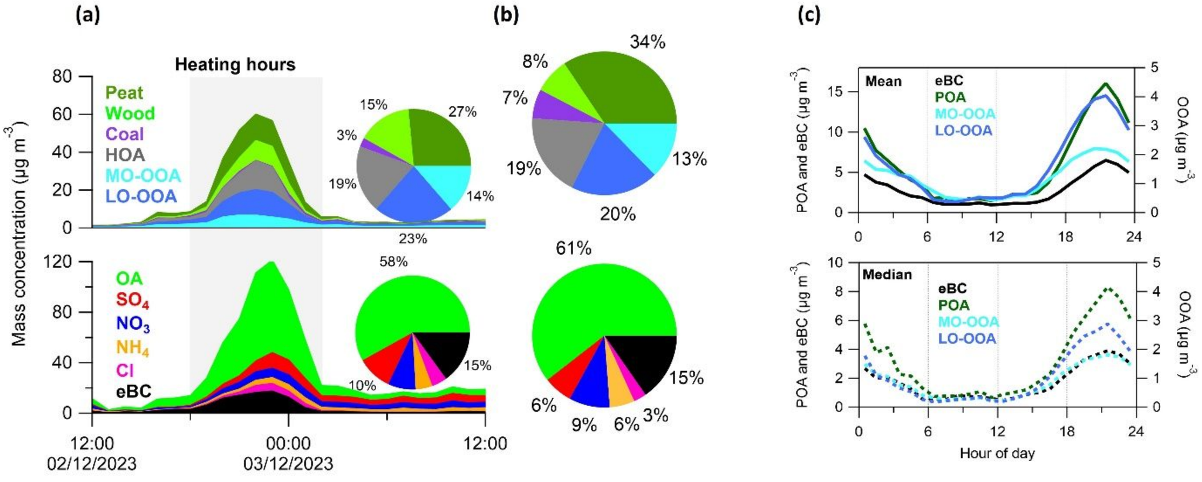

Figure S8. (a) time series of PM_1 species and OA factors during a typical local pollution events in Dublin, with the average chemical composition of PM_1 and OA showing by pie charts. Panel (b) shows the average chemical composition of total PM_1 and OA of all selected unmixed local

events. Panel (c) depicts the diurnal patterns of POA, eBC and two OOA factors during selected local events.

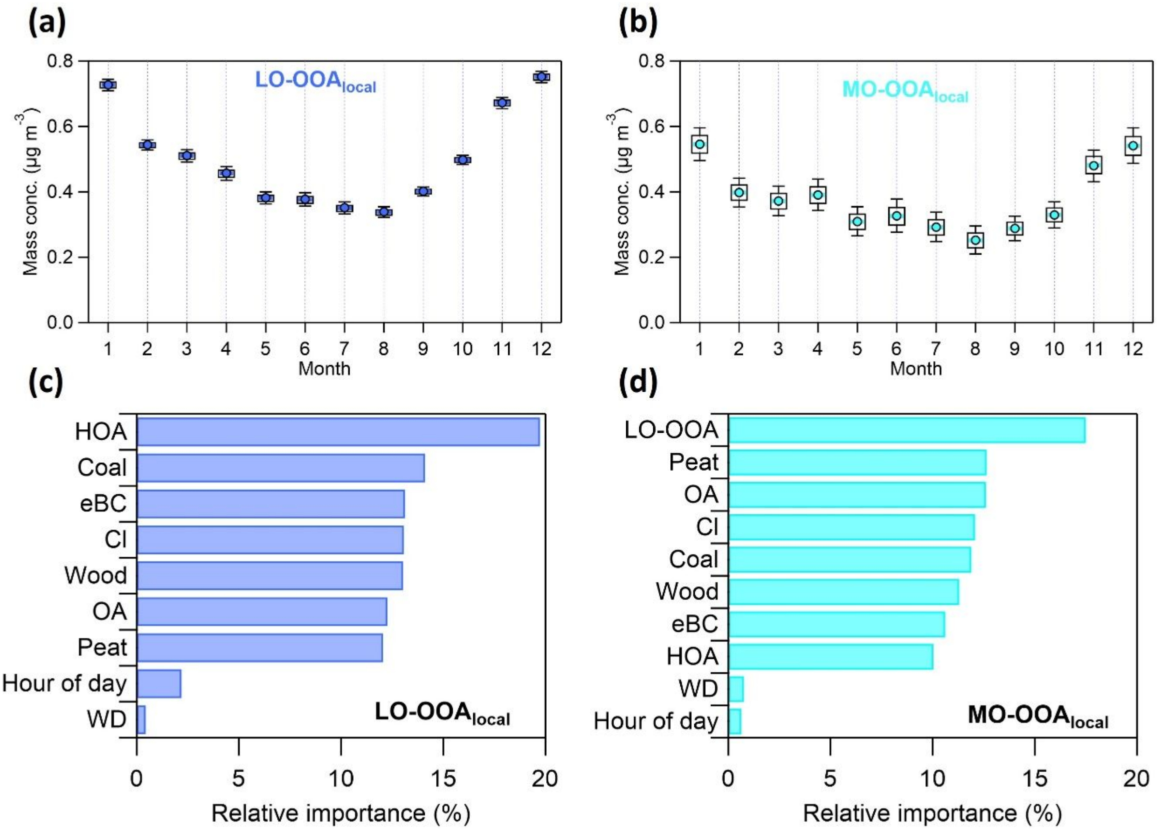

Figure S9. Monte Carlo uncertainty analysis of (a) LO-OOA$_{local}$ and (b) MO-OOA$_{local}$ training results by randomly omitting 20% of the training data and repeating this process for 1000 times. The mean (dots), median (horizontal lines), 25th and 75th percentiles (boxes), and 10th and 90th percentiles (whiskers) of OOA$_{local}$ are shown. The relative permutation importance of predictors on the model predictions of (a) LO-OOA$_{local}$ and (b) MO-OOA$_{local}$ are also shown.

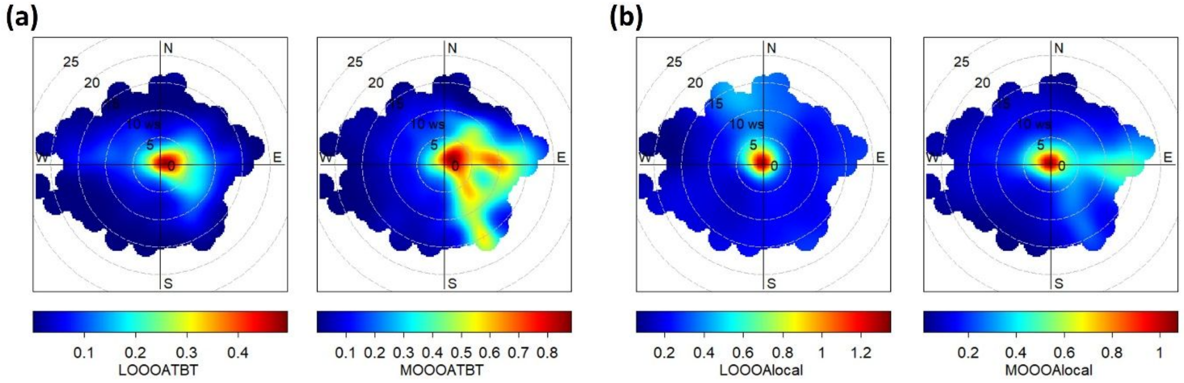

Figure S10. Bivariate polar plots of (a) OOA_{TBT} and (b) OOA_{local}.

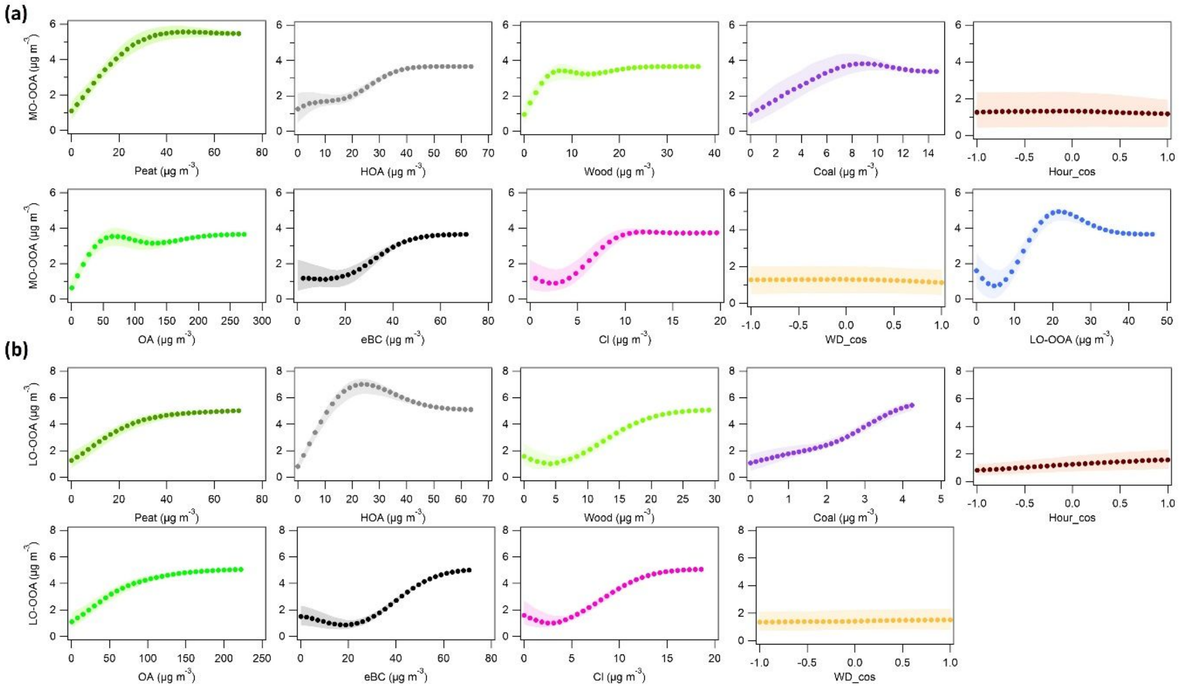

Figure S11. The partial dependences of the model predicted (a) $LO\text{-}OOA_{local}$ and (b) $MO\text{-}OOA_{local}$ on their model predictors. The dots show the median values, while the shaded areas represent the 25th and 75th percentiles.

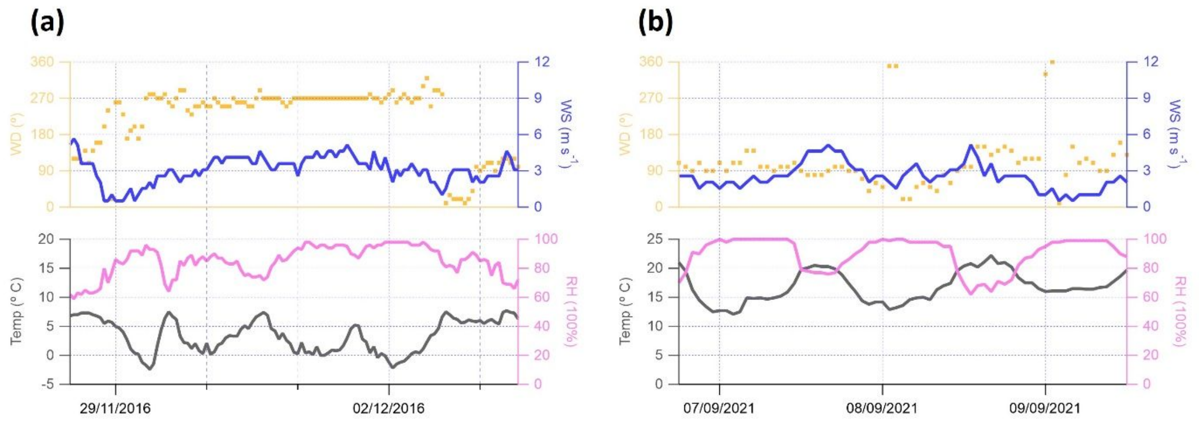

Figure S12. Time series of meteorological parameters, including WS, WD, RH and T during (a) local emission-dominated pollution episode in 2016 and (b) transboundary transport-dominated pollution episode in 2021.

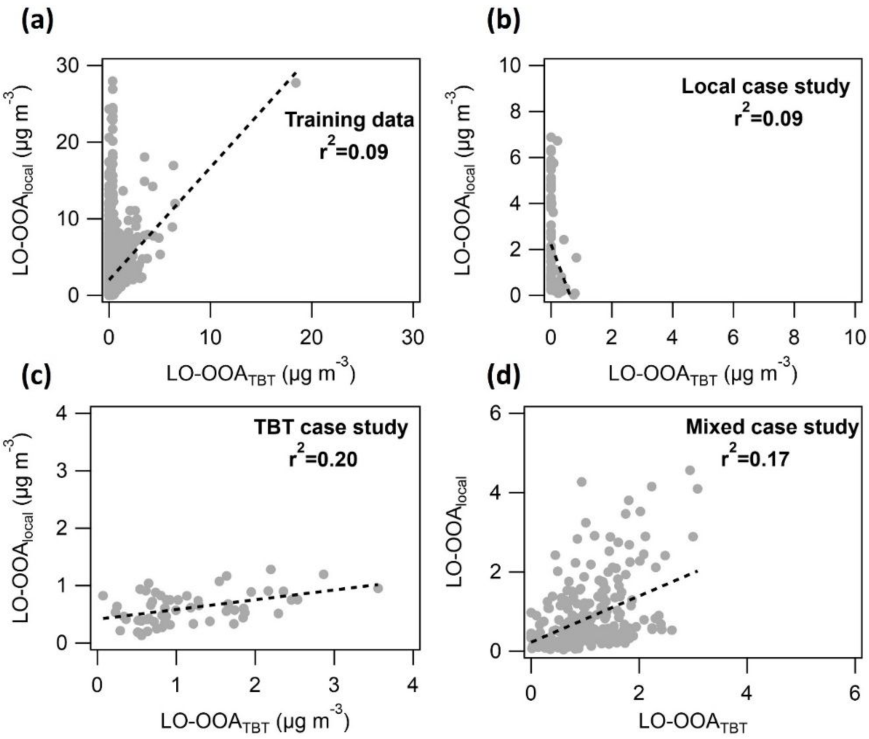

Figure S13. Scatter plots showing the correlation between LO-OOA_{local} and LO-OOA_{TBT} for (a) the training dataset and the selected (b) local (c) transboundary, and (d) mixed event.

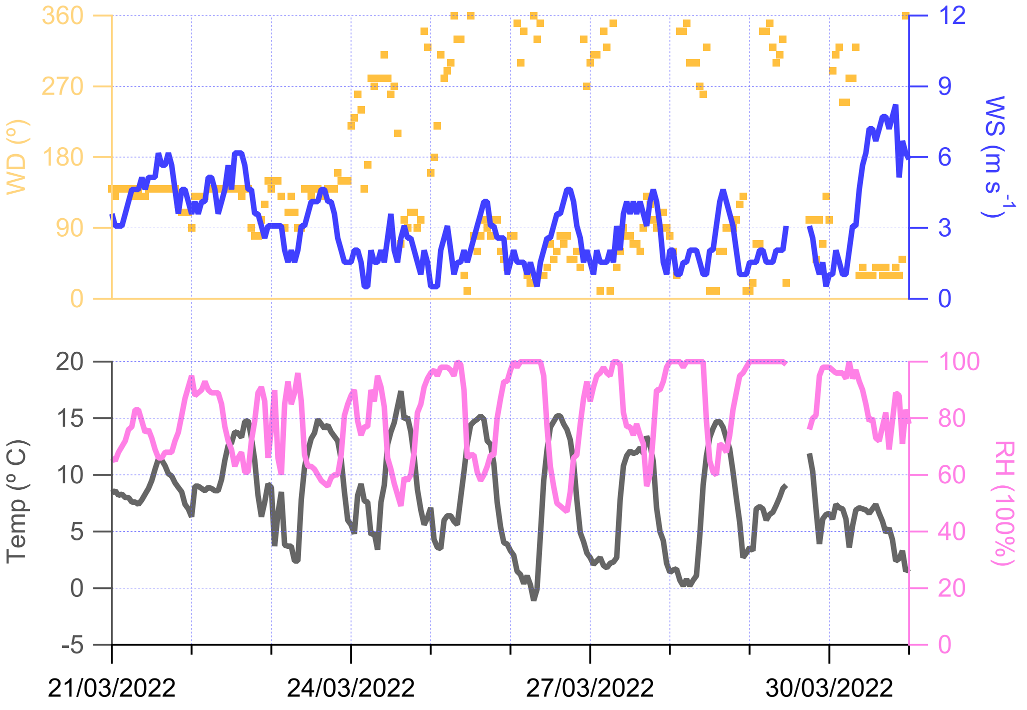

Figure S14. Time series of meteorological parameters, including WS, WD, RH and *T* during the mixed pollution episode in March 2022.

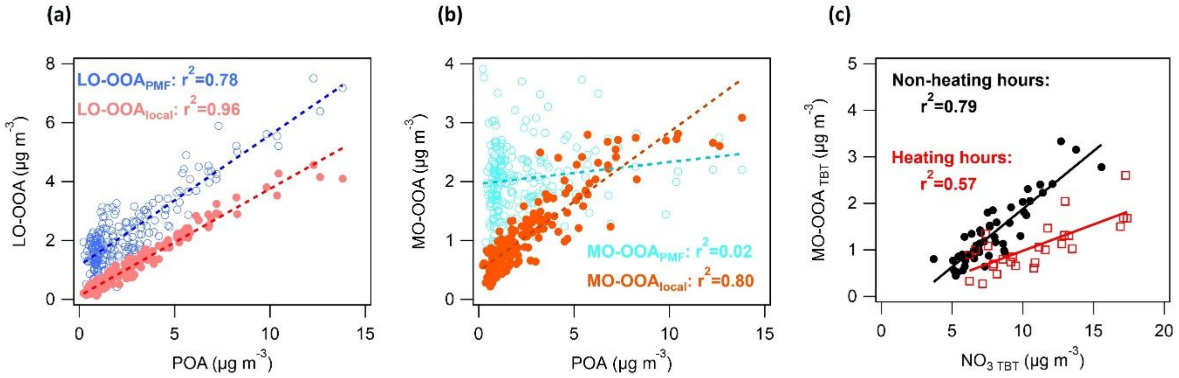

Figure S15. Scatter plots showing the correlation between (a) PMF-derived LO-OOA (LO-OOA$_{PMF}$) and POA versus LO-OOA$_{local}$ and POA, and (b) PMF-derived MO-OOA (MO-OOA$_{PMF}$) and POA versus MO-OOA$_{local}$ and POA during the selected mixed pollution episode in March 2022. Panel (c) shows the correlation between MO-OOA$_{TBT}$ and $NO_{3\ TBT}$ during both non-heating hours (POA < 1.5 μg m^{-3}) and heating hours within the mixed pollution episode.

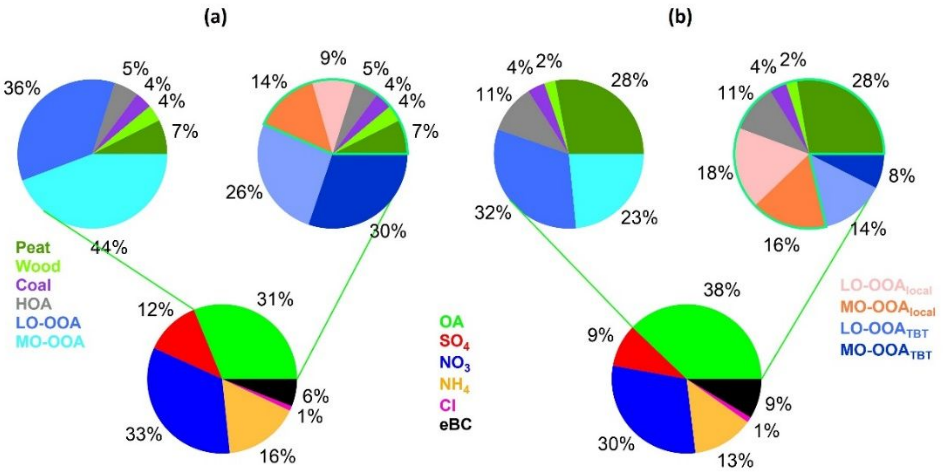

Figure S16. Pie charts showing the average chemical composition of PM_1 and OA during both (a) non-heating (POA < 1.5 μg m^{-3}) and (b) heating hours within the mixed pollution episode in March 2022. The pie charts on the left-top present the average OA composition from PMF analysis, while the pie charts on the right-top shows the average OA composition based on the model prediction with local emissions outlined by green.

REFERENCE

1. Freney, E.; Zhang, Y.; Croteau, P.; Amodeo, T.; Williams, L.; Truong, F.; Petit, J.-E.; Sciare, J.; Sarda-Esteve, R.; Bonnaire, N.; Arumae, T.; Aurela, M.; Bougiatioti, A.; Mihalopoulos, N.; Coz, E.; Artinano, B.; Crenn, V.; Elste, T.; Heikkinen, L.; Poulain, L.; Wiedensohler, A.; Herrmann, H.; Priestman, M.; Alastuey, A.; Stavroulas, I.; Tobler, A.; Vasilescu, J.; Zanca, N.; Canagaratna, M.; Carbone, C.; Flentje, H.; Green, D.; Maasikmets, M.; Marmureanu, L.; Minguillon, M. C.; Prevot, A. S. H.; Gros, V.; Jayne, J.; Favez, O., The second ACTRIS inter-comparison (2016) for Aerosol Chemical Speciation Monitors (ACSM): Calibration protocols and instrument performance evaluations. *Aerosol Science and Technology* **2019,** *53* (7), 830-842.
2. Canonaco, F.; Crippa, M.; Slowik, J. G.; Baltensperger, U.; Prévôt, A. S. H., SoFi, an Igor based interface for the efficient use of the generalized multilinear engine (ME-2) for source apportionment: application to aerosol mass spectrometer data. *Atmospheric Measurement Techniques Discussions* **2013,** *6* (4), 6409-6443.
3. Ulbrich, I. M.; Canagaratna, M. R.; Zhang, Q.; Worsnop, D. R.; and Jimenez, J. L., Atmospheric Chemistry and Physics Interpretation of organic components from Positive Matrix Factorization of aerosol mass spectrometric data *Atmospheric Chemistry and Physics* **2009,** *9*, 2891–2918.

4. Lin, C.; Ceburnis, D.; Trubetskaya, A.; Xu, W.; Smith, W.; Hellebust, S.; Wenger, J.; O'Dowd, C.; Ovadnevaite, J., On the use of reference mass spectra for reducing uncertainty in source apportionment of solid-fuel burning in ambient organic aerosol. *Atmospheric Measurement Techniques* **2021,** *14* (10), 6905-6916.
5. Crippa, M.; DeCarlo, P. F.; Slowik, J. G.; Mohr, C.; Heringa, M. F.; Chirico, R.; Poulain, L.; Freutel, F.; Sciare, J.; Cozic, J.; Di Marco, C. F.; Elsasser, M.; Nicolas, J. B.; Marchand, N.; Abidi, E.; Wiedensohler, A.; Drewnick, F.; Schneider, J.; Borrmann, S.; Nemitz, E.; Zimmermann, R.; Jaffrezo, J. L.; Prévôt, A. S. H.; Baltensperger, U., Wintertime aerosol chemical composition and source apportionment of the organic fraction in the metropolitan area of Paris. *Atmospheric Chemistry and Physics* **2013,** *13* (2), 961-981.
6. Canonaco, F.; Tobler, A.; Chen, G.; Sosedova, Y.; Slowik, J. G.; Bozzetti, C.; Daellenbach, K. R.; El Haddad, I.; Crippa, M.; Huang, R.-J.; Furger, M.; Baltensperger, U.; Prévôt, A. S. H., A new method for long-term source apportionment with time-dependent factor profiles and uncertainty assessment using SoFi Pro: application to 1 year of organic aerosol data. *Atmospheric Measurement Techniques* **2021,** *14* (2), 923-943.
7. Lin, C.; Ceburnis, D.; O'Dowd, C.; Ovadnevaite, J., Seasonality of Aerosol Sources Calls for Distinct Air Quality Mitigation Strategies. *Toxics* **2022,** *10* (3), 121.
